# Robust set-point regulation for ecological models with multiple management goals

**DOI:** 10.1007/s00285-015-0919-7

**Published:** 2015-08-05

**Authors:** Chris Guiver, Markus Mueller, Dave Hodgson, Stuart Townley

**Affiliations:** Environment and Sustainability Institute, College of Engineering Mathematics and Physical Sciences, University of Exeter, Penryn Campus, Penryn, Cornwall TR10 9FE UK; Centre for Ecology and Conservation, College of Life and Environmental Sciences, University of Exeter, Penryn Campus, Penryn, Cornwall TR10 9FE UK

**Keywords:** Population ecology, Resource management, PI control, Positive state system, Anti-windup control, 93C55, 93D15, 93B03, 92D25, 92D40

## Abstract

Population managers will often have to deal with problems of meeting multiple goals, for example, keeping at specific levels both the total population and population abundances in given stage-classes of a stratified population. In control engineering, such set-point regulation problems are commonly tackled using multi-input, multi-output proportional and integral (PI) feedback controllers. Building on our recent results for population management with single goals, we develop a PI control approach in a context of multi-objective population management. We show that robust set-point regulation is achieved by using a modified PI controller with saturation and anti-windup elements, both described in the paper, and illustrate the theory with examples. Our results apply more generally to linear control systems with positive state variables, including a class of infinite-dimensional systems, and thus have broader appeal.

## Introduction

Regulation by feedback arises in numerous areas of science and engineering; such as acoustics, electrical circuits, aviation and biological systems. According to the report of Murray et al. (2003): “Feedback is an enabling technology in a variety of application areas and has been reinvented and patented many times in different contexts”. Ubiquitous to the design and synthesis of modern feedback control systems are (P)roportional, (I)ntegral, (D)erivative controllers. These dynamical models incorporate current (P part), past (I part) and predictive (D part) information about a measured variable or variables and create from this information a signal, termed an input or control, which is then fed back into the to-be-controlled system to achieve some desired dynamic behaviour. PID controllers are widely used in industrial processes (Lunze 1989; Åström and Hägglund 1995) and have been described as one of the “Success Stories in Control” (Samad and Annaswamy 2011, p. 103). The special case of integral control was developed in the 1970s as a technique for regulating the measured variables of a stable, but controlled, linear system to a fixed and chosen set-point. Early contributions to the theory of proportional and integral (PI) control are found in the control engineering literature and include Davison (1975, 1976), Lunze (1985), Morari (1985) and Grosdidier et al. (1985). Whilst grounded in the field of process engineering, applications of PI control are multiple and varied. Indeed, established examples in engineering are complemented by emerging examples in biology, such as the regulation of blood sugar by insulin (Saunders et al. 1998), bacterial chemotaxis in living cells (Yi et al. 2000), calcium homeostasis (El-Samad et al. 2002) and, recently in Guiver et al. (2015), ecological management—the continued focus of the present work.

In ecological management, PI control provides a suite of techniques for management by the addition or removal of individuals from an ecological process, such as a population. In applied contexts, addition may correspond to captive-release schemes, translocation or replanting and removal may correspond to harvesting, culling or coppicing. Consequently, applications of PI control are broad in scope and importance, including pest or resource management, agriculture, horticulture and conservation. Its scope potentially extends to key and immensely timely societal challenges of the twenty-first century, such as food security (Godfray and Garnett 2014). Indeed, UNESCO’s Mathematics of Planet Earth’s 2013 programme^1^ was “born from the will of the world mathematical community to learn more about the challenges faced by our planet and the underlying mathematical problems, and to increase the research effort on these issues” including “[a] growing population competing for the same global resources”. In addition to the potential applications, our motivation for exploring the utility of PI control in ecological management is twofold: (a) their ease of computation and implementation, with very little knowledge required of the to-be-controlled system, and; (b) their inherent robustness to various forms of uncertainty. We further elaborate on (a) and (b) in the manuscript and contend that these facets make PI control ideally suited for ecological management where processes are subject to unknown disturbances and dynamic models are (possibly) highly uncertain. The PI controllers that we propose here do not seek to use measured data to update the underlying ecological model over time, by inferring parameters for instance, but the control does change in response to a measured variable. In this sense and context, feedback control has parallels to adaptive management, an approach well known in the resource and ecological management literature (Holling 1978; Walters 1986; Williams 2011). Other authors have noted this connection as well: Heinimann (2010) proposes principles from control theory as a concept for scholars and practitioners in adaptive ecosystem management.

Our earlier paper, Guiver et al. (2015), introduces integral control and PI control, in a context of single management goals, for structured population models. These deterministic population or meta-population models stratify individuals according to some discrete or continuous age-, size- or stage-structure and include matrix (P)opulation (P)rojection (M)odels (Caswell 2001; Cushing 1998) and (I)ntegral (P)rojection (M)odels (Easterling et al. 2000; Ellner and Rees 2006; Briggs et al. 2010). Guiver et al. (2015) considers regulation of single (scalar) observations or measurements to a prescribed set-point, or, in ecological modelling parlance, achieves a single management goal or objective. It is reasonable to request, however, that more than one measurement is regulated, and that more than one per time-step management action is permitted. For example, when designing a replanting programme to conserve a declining plant population, regulating total abundance may not be as beneficial as thought when the composition of the resulting stratified population is dominated by the seed stage-class. It may be more desirable to control *both* total abundance *and* abundance of a given stage-class, for instance, flowering plants. Alternatively, in sustainable harvesting, it may be desirable to harvest (that is, remove from) certain stage-classes whilst replenishing others, and still maintain a desired abundance of certain stages.

The application of PI control to the above multi-objective management problem is novel itself and, we believe, a useful and timely contribution to the suite of tools available to population managers, conservation biologists and other end users. To present such a solution requires new mathematical results in control theory for two reasons. First, population level models, such as matrix PPMs and IPMs, are examples of positive dynamical systems and existing “off-the-shelf” PI control need not respect the necessary nonnegativity constraints. In an applied context the controller could instruct management actions that are counter-intuitive or, worse, meaningless, such as removing more individuals than are currently present. Second, when the measured-variables are naturally constrained to be nonnegative, it is clear that not every nonnegative vector with more than one component is a feasible set-point. For example, if one measurement is always required to be larger than another, then this ordering must be preserved in the candidate set-point, as is the case in the plant example alluded to above.

Therefore, in the present contribution we apply low-gain PI control with multiple management goals (so-called multi-input, multi-output systems) to examples in ecological management and develop low-gain PI control for discrete-time, positive state linear systems. The models and terminology is further explained throughout the manuscript. The material we present is an extension of Guiver et al. (2015), where an existing suite of results in control theory was drawn upon and further developed to address the nuanced situation of positive state variables and input constraints. Analogously, in regulating multiple outputs with multiple saturating inputs, we need to develop a different set of tools, described in the manuscript, and in particular draw upon recent positive state results in Guiver et al. (2014). Our results apply to situations outside of ecology, adding to their appeal, and are novel, although there are similarities to the results of Nersesov et al. (2004). We compare and contrast their approach with ours in Remark 4.8.

Owing to its dual focus, the manuscript has the following deliberate structure. Section 2 seeks to further motivate PI control as a tool for ecological management, informally states our main result and illustrates its application through an example. The subsequent Sects. 3 and 4 form the technical heart of the manuscript and develop the mathematics summarised in Sect. 2. In order to extend the appeal of this contribution, including to a possibly non-mathematical audience, we have deliberately placed proofs of all novel results in “Appendix C”. A second example is presented in Sect. 5 and the manuscript is concluded by Sect. 6 with a discussion. “Appendices A and B” contain model parameters used in the examples that are not given in the main text for ease of presentation and preliminary material required for the proofs of our results, respectively.

## Motivation, main result and illustrative example

This section contains an informal overview of our main results and demonstrates their possible application. We seek as well to further motivate the present contribution by briefly discussing the distinction between *robust* and *optimal* control, particularly in the context of ecological management. A larger, more comprehensive, introduction to PI control in the same context is contained in our earlier manuscript (Guiver et al. 2015, Section 2) which, to avoid repetition, we have not reproduced fully here. We mention that Guiver et al. (2015, Section 2.1) compares and contrasts PI control with other theoretical approaches to ecological management available in the literature.

For the situation considered here, the key ingredients are:

- a managed population or resource that is changing over time (referred to as the to-be-controlled system or just system);
- the possibly disturbed observations or measurements (referred to as outputs);
- a management strategy that permits the addition or removal of individuals (referred to as control actions).

The outputs provide information about aspects of the population, say abundance of a strata, and the present PI control problem is to choose a series of control actions to subsequently manage these outputs, that is, to regulate them to prescribed quantities. A PI controller is, in essence, a mathematical model that uses functions of the measurements to determine present and future control actions.

To describe PI control, a model of the to-be-controlled system is required. We shall assume that the population is modelled by a deterministic, linear, stratified population model, typically a matrix PPM (Caswell 2001). PPMs are structured population models, meaning that the modelled population is partitioned into discrete age-, size- or developmental stage-classes (the latter may include larval, pupal, adult, etc.). A linear, time-invariant matrix PPM is given by

2.1 $$\begin{aligned} x(t+1) = A x(t), \quad x(0) = x^0, \quad t = 0,1,2,\dots , \end{aligned}$$

where *x*(*t*) denotes the structured population, in integer *n* stage-classes, with initial population distribution $x^0$ and *A* is an $n \times n$ componentwise nonnegative matrix. The time-steps *t* in (2.1) are assumed fixed: a week, month, or breeding cycle, for instance. The matrix *A* in (2.1) is often called the projection matrix, and contains life-history parameters of the population, such as recruitment, survival and transitions between stage-classes.

The inclusion of measurements *y*(*t*) and control actions *u*(*t*) in (2.1) leads to the model

2.2 $$\begin{aligned} \left. \begin{aligned} x(t+1)&= A x(t) + B u(t),&x(0)&= x^0\,, \\ y(t)&= C x(t),&\end{aligned} \right\} \quad t =0,1,2,\dots \,, \end{aligned}$$

where the input vector *u*(*t*) with integer *m* components is to-be-determined by the modeller. The terms *B* and *C* in (2.2) are $n \times m$ and $p \times m$ matrices, respectively, where *p* denotes the number of per time-step measurements taken. We note that, at any given time-step *t*, the entire population distribution (the state) *x*(*t*) may not be known (or known precisely), and consequently may not be used to help determine *u*(*t*). This is not necessarily a problem for feedback control—as we explain in Sect. 4.4.1, knowledge of *x*(*t*) is not required for PI control to succeed (knowledge of the matrix *A* is not required either) and PI control provides so-called global results in that they hold for any initial population distribution $x^0$. What is crucial to the efficacy of feedback control is access to the measured variable *y*(*t*). The key difference between the present contribution and Guiver et al. (2015) is that in the latter we restricted attention to $m=p=1$ but here the situation $m,p>1$ is permitted, implying that numerous measurements are recorded and management actions taken—so-called management with multiple goals in ecological terminology or the multi-input, multi-output case in control theoretic terminology.

Matrix PPMs (2.1) are examples of discrete-time, positive dynamical systems—“positivity” refers to the property that the state-variables take only nonnegative values, typically denoting abundances, densities or concentrations. Positive dynamical systems form the appropriate framework for a variety of physically meaningful mathematical models and arise as models in a diverse range of fields from biology, chemistry, ecology and economics to genetics, medicine and engineering (Haddad et al. 2010, p. xv). Owing to their importance in mathematical modelling positive dynamical systems are well-studied with textbooks by, for example, Berman et al. (1989), Krasnosel’skij et al. (1989) and Berman and Plemmons (1994). The theory of *linear* positive dynamical systems is rooted in the seminal works of Perron (1907) and Frobenius (1912) on nonnegative matrices (for a recent treatment see, for example, Berman and Plemmons 1994, Chapter 2). Control of positive dynamical systems leads to positive input control systems (Farina and Rinaldi 2000), where the input variables are also assumed to be positive. Presently, only the state *x*(*t*) and output *y*(*t*) in (2.2) need take componentwise nonnegative values, so called so-called positive state systems (Guiver et al. 2014). Accordingly, *u*(*t*) may take negative values, provided that a nonnegative number or distribution remains. Such a framework allows the modelling of control actions (or disturbances) such as harvesting, culling, pest management or predation; actions which, importantly, fall *outside* the existing positive systems theory.

As a concrete and illustrative example, we explore the potential utility of low-gain PI control by applying it to the management of a pronghorn (*Antilocapra americana*) population based on matrix models from Berger and Conner (2008). Pronghorn are native to Canada, Mexico and the US, and currently occur in western North America from Canada through to northern Mexico. Managed populations are found in Yellowstone National Park and across the continent numbers are generally stable, having recovered from near extinction in the 1920s. The species is susceptible, however, to habitat loss from urban and agricultural expansion and restriction of seasonal movements from fencing (Hoffmann et al. 2008). Pronghorn is legally hunted with permits, although the subspecies Sonoran pronghorn is endangered and populations in Arizona and Mexico are protected under the US Endangered Species Act.

The example also seeks to highlight the drawbacks of “off-the-shelf” PI control in this particular applied context and to motivate additional novel features we develop in Sect. 4. The pronghorn projection matrix model is an age-structured model, with time-steps denoting years, and is based on Berger and Conner (2008, Table 4, wolf-free site). The models provided there are for female pronghorn, although presently we have included males in the population as well. Consequently there are six stage-classes denoting female and male, neonates, yearlings and prime adults. The model parameters used may be found in “Appendix A”. The spectral radius of the projection matrix *A* in (2.1) or (2.2) is $\lambda = 0.9222 <1$ so that the uncontrolled population *x*(*t*) specified by (2.1) [or (2.2) with $u(t) =0$ for every *t*] is declining asymptotically. Suppose, therefore, that the hypothetical management objectives are to raise abundance of female and male prime adults to 120 and 100, respectively, from their initial abundances of c. 95 and c. 30, assuming a total initial population abundance of 300. In order to be regulated to the chosen set-point

2.3 $$\begin{aligned} r = \begin{bmatrix}120 \\100 \end{bmatrix}, \end{aligned}$$

the female and male prime adults stage-classes must be observed each time-step, determining the matrix *C* in (2.2). To affect these changes at least two per-time step management actions (the same number as observations) are required, and we assume that we may replenish female and male neonates, determining *B* in (2.2). The first and second component of the vector-valued input variable *u*(*t*) in (2.2) now denote how many female and male individuals are released per time-step, respectively. The first difficulty to overcome is to determine, given the particular *A*, *B* and *C* specified by the pronghorn model, whether it is possible to choose an input *u*(*t*) such that the output *y*(*t*) does indeed converge to *r* in (2.3)? Note that the state and output variables must remain nonnegative for a meaningful model and this nonnegativity requirement in turn imposes geometric constraints on the set of possible inputs *u*(*t*). For the sequel we record this problem as:

(P1): which nonnegative set-points can be tracked asymptotically whilst preserving nonnegative state and output variables?

Informally, we say that set-points that may be asymptotically tracked with nonnegative state and output variables are feasible and we demonstrate in the “Appendix A” that the set-point *r* in (2.3) is indeed feasible.

**Fig. 1:**
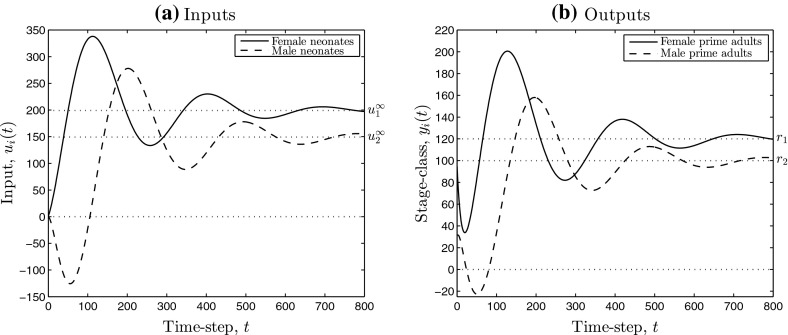
Simulations of the low-gain integral control model (I) (see Sect. 3.2) applied to the pronghorn matrix model. **a**, **b** Contain the inputs and outputs, respectively. The *dotted lines* denote the components of the limiting input (**a**) and the chosen set-point (**b**)

Figure 1 shows simulation results obtained by applying low-gain integral control to the pronghorn model. Although the output, here denoting measured abundance of each stage-class, converges to the chosen set-point *r* over time, four deficiencies of the “off-the-shelf” integral controller are demonstrated:

(i) during time-steps 50–150, substantially more than 200 female neonates must be added to the population per time-step (Fig. 1a), which may be too large to be practical;
(ii) during the same time-steps, the integral controller is instructing the removal of male neonates, that is, $u_2(t) <0$ (Fig. 1a), which seems unnecessary and wasteful;
(iii) most crucially, the resulting measurements $y_2(t)$ of male prime adults are negative for some *t* (Fig. 1b), which is absurd for this model, and;
(iv) the performance is very slow, predicting at least 500 years(!) to converge to the desired set-point.

Whilst we acknowledge that the model parameters have been chosen somewhat pathologically to emphasise these deficiencies, they do help motivate the present contribution quite markedly.

Deficiencies (i)–(iii) above are addressed by considering a modified low-gain PI control model that includes input saturation. In words, negative inputs *u*(*t*) (that is, when the management strategy suggests removal of individuals) are replaced by zero and a per time-step maximum bound is imposed for *u*(*t*), reflecting limited per time-step resources or management capability. As we explain in Sect. 4, doing so introduces a nonlinearity into the feedback model and establishing convergence of the output to the set-point is more challenging. For the sequel, we record:

(P2): how can input saturation be included in low-gain PI control and still ensure that the desired set-point is tracked asymptotically by the output?

**Fig. 2:**
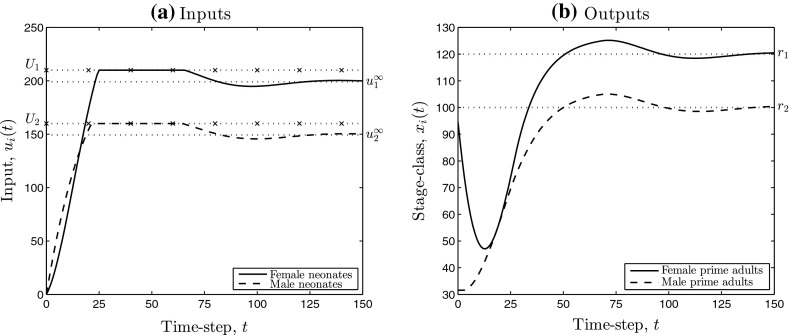
Simulations of the low-gain integral control model (Iaw) (see Sect. 4.2) applied to the pronghorn matrix model. **a**, **b** Contain the inputs and outputs, respectively. The *dotted lines* denote the components of the limiting input (**a**) and the chosen set-point (**b**). The *dotted–crossed lines* in **a** denote per time-step input saturation limits

Deficiency (iv) of rate of convergence of the feedback model may be adjusted by the use of a (P)roportional component as well as an (I) component, as we describe in the manuscript. Our main results are low-gain PI control models for positive state linear systems that address issues (P1) and (P2), stated as Theorem 4.6 and Corollary 4.7. We establish several robustness results in Sect. 4.4, that capture how the low-gain PI control systems can handle uncertainty. Figure 2 contains simulation results obtained by applying low-gain integral control with input saturation to the pronghorn model. From the simulations we see that each of issues (i)–(iv) present in Fig. 1 do not appear and a robust solution to the stated management problem is provided.

Having outlined a low-gain PI control solution to the above management problem, we comment on how the solution may be additionally combined with other management approaches present in the literature and, moreover, how feedback control differs from optimal control. These latter observations are intended to further motivate the present exploration of the utility of PI controllers in ecological management.

### *Remark 2.1*

(i) An existing suite of management strategies proposed in ecological matrix modelling are based on tools from perturbation theory. Typically, modelled vital rates are altered with a view to obtaining some asymptotically desired dynamic behaviour (such as stasis or growth in conservation), which is described by replacing *A* in (2.1) with $A + {\Delta }$, for some perturbation matrix ${\Delta }$. Sensitivity (Demetrius 1969) or elasticity (Kroon et al. 1986) analyses are often employed and use methods from calculus to determine the effect of small changes in particular vital rates on the resulting asymptotic behaviour. These calculations are used to inform where potential management or conservation strategies should invest their efforts. Numerous examples are present in the literature and we highlight, for example, shark conservation (Otway et al. 2004) and the effects of Brazil nut tree seed extraction on its demography (Zuidema and Boot 2002). Biologically, perturbation analysis denotes improving or degrading vital rates through environmental or demographic changes, the former for instance, through improved quality or access to food or decreased mortality rates by protecting habitats. These methods are not directly comparable to PI control, as they do not denote the addition or removal of individuals, but may be combined with PI control approaches. We revisit the above pronghorn example in Sect. 5 and combine low-gain PI control proposed here with a second management strategy.
(ii) Low-gain PI control is an example of feedback control. Complementary to feedback control is optimal control which, to some audiences, may be synonymous with control theory itself. Here an input is chosen to achieve some desired dynamic behaviour as well as to minimise a prescribed functional; typically denoting the cost or effort of the management strategy in ecological applications. Optimal control has proven very popular in mathematical biology (Lenhart and Workman 2007). Pontryagin’s celebrated maximum principle (see, for example, Liberzon 2011, Chapter 4) has been employed in models for the optimal control of HIV (Kirschner et al. 1997), epidemics (Hansen and Day 2011) and vector-borne diseases (Blayneh et al. 2009). Techniques from optimal control have appeared extensively in the mathematical ecology, conservation and resource management literature where an input to a control system denotes a management strategy that is applied to a ecological process, such as a modelled population. To name but a few examples, research by Hastings and collaborators has tackled optimal management of deterministic models for the invasive perennial deciduous grass *Spartina* by applying linear programming (Hastings et al. 2006), so-called linear quadratic optimal control (Blackwood et al. 2010) or dynamic programming (Lampert et al. 2014). Elsewhere applications of Pontryagin’s maximum principle have appeared in the fisheries management literature (Kellner et al. 2011; Moeller and Neubert 2013). Solutions to population management problems have also been proposed by optimising prescribed cost-functionals in the situation when the underlying dynamics are assumed stochastic, such as those given by (P)artially (O)bservable (M)arkov (D)ecision (P)rocesses (Monahan 1982). Stochastic dynamic programming techniques are then used to numerically compute optimal strategies. Substantial research has been undertaken by Possingham and collaborators, including Shea and Possingham (2000), Chadès et al. (2011) and Regan et al. (2011).

Whilst the design of management strategies via optimal control have an appeal in that they would minimise some specified cost, there are downsides. First, computing optimal controls is often analytically intractable or computationally highly expensive [suffering from, for example, the “curse of dimensionality”, coined in Bellman (1957), see more recently Powell (2007)] and so optimal controls can be impractical to implement. Second, and often overlooked, it is not always clear that “off-the-shelf” optimal control approaches will respect positivity of the system states (although one exception we are aware of appearing in the control literature is Nersesov et al. (2004)). Third, and a more serious and pressing obstacle, population-level ecological models are typically highly uncertain. Uncertainty is a broad term in ecology and ecological modelling, although in this context both Regan et al. (2002) and Williams (2001) contain helpful and interesting codices of the term. Presently, uncertainty encompasses choice of model structure (for example, type of model, number of stage-classes or any modelled density-dependence), parametric uncertainty (for instance, how to accurately fit vital rates for a chosen model) and unknown disturbances of the dynamics (such as unmodelled immigration or sampling error). Therefore, we argue that it is essential that ecological management strategies, be it for sustainable harvesting, pest management or conservation, are designed to be robust. Informally, a control scheme is robust with respect to a source of uncertainty if it performs as intended in spite of that uncertainty. Another facet of robust control is quantifying the extent to which a control objective fails when operating in uncertain or unknown operating conditions. The study of robust control [with textbooks by, for example, Green and Limebeer (1995) or Zhou and Doyle (1998)] was in part born out of the hugely important observation by control engineers in the 1970s that optimal control techniques need *not be* robust and, moreover, over-optimisation leads to fragility (Doyle 1978). Indeed, as we sought to emphasise in Guiver et al. (2015), so-thought optimal controls can have disastrous performance when applied to an uncertain model and hence our current continued exploration of robust feedback control in ecological management.

## Problem formulation: multi-input, multi-output low-gain PI control

Sections 3 and 4 contain the technical heart of the manuscript where we formulate both the problem exposited in Sect. 2 and its solution. Specifically, in this section we recap so-called multi-input, multi-output low-gain PI control and in the next we extend known results to address the issues (P1) and (P2). Recall that proofs of all novel stated results are contained in “Appendix C”.

### Notation

We introduce some notation, although most notation we use is standard, or is defined as it is introduced. Briefly, we let $\mathbb {N}_0$, $\mathbb {N}$, $\mathbb {R}$ and $\mathbb {C}$ denote the sets of nonnegative integers, positive integers, real and complex numbers, respectively. For positive integer *n*, denoted $n \in \mathbb {N}$, we let $\mathbb {R}^n$ and $\mathbb {C}^n$ denote real and complex *n*-dimensional Euclidean space, respectively, equipped with the usual two-norm, always denoted by $\Vert \cdot \Vert$. As usual, we let $\mathbb {R}^1 = \mathbb {R}$ and $\mathbb {C}^1 = \mathbb {C}$. For $m \in \mathbb {N}$, $\mathbb {R}^{n \times m}$ and $\mathbb {C}^{n \times m}$ denote the sets of $n \times m$ matrices with real and complex entries, respectively. We shall denote by *I* the identity matrix, used consistently without specifying its dimensions. The notation $\Vert \cdot \Vert$ also denotes the operator two-norm induced from $\Vert \cdot \Vert$ on $\mathbb {C}^n$ or $\mathbb {R}^n$. We denote by *r*(*A*) the spectral radius of $A \in \mathbb {C}^{n \times n}$ which, recall, is given by

$$\begin{aligned} r(A) = \sup \{ \vert \lambda \vert :\lambda \in \sigma (A)\,\}, \end{aligned}$$

where $\sigma (A)$ denotes the spectrum of *A*—its set of eigenvalues when *A* is a matrix. The state *x* of the uncontrolled linear model (2.1) converges to zero or diverges to infinity when $r(A) <1$ or $r(A) >1$, respectively (the latter at least for some nonzero initial states $x^0$).

The symbols $\mathbb {R}_+^n$ and $\mathbb {R}^{n \times m}_+$ denote the sets of componentwise nonnegative vectors and matrices, respectively. A vector *z* in $\mathbb {R}^n$ belongs to $\mathbb {R}^n_+$ if $z_k \ge 0$ for every *k*, where $z_k$ denotes the $k{\mathrm{th}}$ component of *z*. We call vectors $z \in \mathbb {R}^n_+$ nonnegative and say that $z \in \mathbb {R}^n_+$ is positive if $z_k >0$ for every *k*. For vector $z \in \mathbb {R}^n$, the term $\Vert z \Vert _1$ denotes the vector one-norm of *z*, and is defined as

$$\begin{aligned} \Vert z\Vert _1 := \sum _{k=1}^n \vert z_k\vert = \sum _{k=1}^n z_k, \quad \text {if }z \hbox { nonnegative.} \end{aligned}$$

The superscript ${}^T$ denotes matrix or vector transposition, so that if $z \in \mathbb {R}^n$ then $z^T$ is a row vector.

### Multi-input, multi-output low-gain PI control

For the most part in the present manuscript we consider the discrete-time linear model (2.2) where

3.1 $$\begin{aligned} (A,B,C) \in \mathbb {R}^{n \times n} \times \mathbb {R}^{n \times m} \times \mathbb {R}^{p \times n}, \end{aligned}$$

for $n,m,p \in \mathbb {N}$ and given $x^0 \in \mathbb {R}^n$. The variables *u*, *x* and *y* denote the input, state and output of (2.2), respectively. Although our motivating applications are the management of ecological models where the input, state and output typically have clear biological interpretations, here we are describing the more general situation. In particular, PI control does not require nonnegativity assumptions on *A*, *B* or *C*. We shall impose additional structure on (2.2) and (3.1) in Sect. 4.

The transfer function *G* of the linear system (2.2) [also of the triple (*A*, *B*, *C*)] is the function of a complex variable, defined as

3.2 $$\begin{aligned} G:\mathbb {C}\rightarrow \mathbb {C}^{p \times m}, \quad z \mapsto G(z) := C(zI - A)^{-1}B, \end{aligned}$$

where recall that *I* in (3.2) is the (here $n \times n$) identity matrix. The function *G* is certainly well-defined for every complex *z* that is not an eigenvalue of *A* and, moreover, provides a relationship between an input *u* and the resulting output *y* related by (2.2). More information about *G* is contained in “Appendix B” but, it suffices here to note that if $r(A) <1$ then *G*(1) is well-defined and has the property that if *u* has a limit $u^\infty$ then, for any initial state $x^0$, *y* in (2.2) has the limit

3.3 $$\begin{aligned} \lim _{t \rightarrow \infty } y(t) = C(I-A)^{-1}B u^\infty = G(1) u^\infty . \end{aligned}$$

From the Neumann series definition

$$\begin{aligned} G(1) = C(I-A)^{-1}B = \sum _{k\in \mathbb {N}_0} CA^k B = C(I + A + A^2 + \cdots )B, \end{aligned}$$

and the limit relationship (3.3) it follows that the $(i,j){\mathrm{th}}$ entry of *G*(1) is the eventual $i{\mathrm{th}}$ measurement when the $j{\mathrm{th}}$ input variable is one for all times. The interpretation is somewhat similar to that of the fundamental matrix in matrix population modelling (Caswell 2001, p. 112). By conducting controlled experiments, such as in applications in electrical circuits, it is sometimes possible to obtain an estimate of *G*(1) (Penttinen and Koivo 1980; Lunze 1985), although this is possibly inappropriate in ecological management.

Integral control has been developed in the situation $r(A)<1$ to solve the so-called set-point regulation problem or objective, namely, to generate an input *u* such that the resulting outputs *y* of (2.2) converge to a prescribed set-point $r \in \mathbb {R}^p$. The objective should be achieved independently of the initial state $x^0$ and with only knowledge of *y* and *G*(1). The internal model principle (Francis and Wonham 1976) dictates that in order to achieve the set-point regulation objective via feedback control, the control strategy must contain an integrator, or synonymously an integral controller which, when connected via feedback to (2.2), leads to: 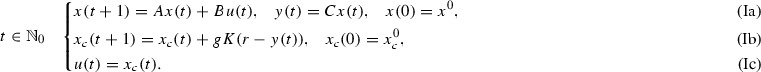 Equation (Ia) is model (2.2)—the to-be-controlled system with the measured output *y*(*t*). Equation (Ib) is the integral controller model, with $x_c(t) \in \mathbb {R}^m$ for each $t \in \mathbb {N}_0$ denoting its state with initial state $x_c^0$. Equation (Ic) is a feedback connection from (Ib) to (Ia) via the input *u*(*t*). The terms $K \in \mathbb {R}^{m \times p}$, $g>0$ and $x_c^0 \in \mathbb {R}^m$ in (Ib) are design parameters and *r* is the desired set-point.

The following “low-gain” result for integral control is well-known and based on, for example, Logemann and Townley (1997, Theorem 2.5, Remark 2.7). The term “low-gain” refers to the fact that the positive parameter *g* in (I) (often called a “gain”) is required to be sufficiently small.

#### **Theorem 3.1**

(Low-gain integral control) Suppose that the integral control system (I) with $m=p$ satisfies

1. $$r(A) <1$$, and;
2. *K* and *G*(1) are such that every eigenvalue of the product *KG*(1) has positive real part.

Then, there exists $g^*>0$ such that for all $g \in (0,g^*)$, all $r \in \mathbb {R}^p$ and all $(x^0, x^0_c) \in \mathbb {R}^n \times \mathbb {R}^m$ the solution $(x,x_c)$ of (I) has the properties:

- $$\displaystyle {\lim \nolimits _{t\rightarrow \infty }}x_c(t)= x_c^\infty : = G(1)^{-1}r$$;
- $$\displaystyle {\lim \nolimits _{t\rightarrow \infty }}x(t)= x^\infty := (I-A)^{-1}BG(1)^{-1}r$$;
- $$\displaystyle {\lim \nolimits _{t\rightarrow \infty }}y(t)= \displaystyle {\lim \nolimits _{t\rightarrow \infty }}Cx(t)= r$$.

When $r(A) \ge 1$ then the conclusions of Theorem 3.1 do not apply to (I). However, in this situation (I) can be modified by including a (P)roportional feedback component. Specifically, the feedback connection (Ic) is replaced by

3.4 $$\begin{aligned} u := -F_1 x + x_c, \end{aligned}$$

if the state *x* is known and available to the modeller, or by

3.5 $$\begin{aligned} u := -F_2y + x_c, \end{aligned}$$

when only the output *y* is available. The matrices $F_1 \in \mathbb {R}^{m \times n}$ and $F_2 \in \mathbb {R}^{m \times m}$ are additional design parameters. We denote by (PI1) and (PI2) the combinations of (Ia), (Ib) and (3.4) or (Ia), (Ib) and (3.5), respectively. For completeness, we record that (PI1) is given by

PI1 $$\begin{aligned} \left. \begin{aligned} x(t+1)&= Ax(t) + Bu(t),&x(0)&= x^0, \\ x_c(t+1)&= x_c(t) + gK(r - Cx(t)),&x_c(0)&= x_c^0, \\ u(t)&=-F_1x(t) + x_c(t)\,,&\end{aligned}\right\} \quad t \in \mathbb {N}_0 \end{aligned}$$

while in (PI2) the third line of (PI1) is replaced by (3.5). Inserting the expression for *u* in (PI1) into the dynamic equation for *x* also in (PI1) and introducing the new input variable $v := x_c$ yields

$$\begin{aligned} \left. \begin{aligned} x(t+1)&= (A-BF_1)x(t) + Bv(t),&x(0)&= x^0, \\ x_c(t+1)&= x_c(t) + gK(r - Cx(t)),&x_c(0)&= x_c^0, \\ v(t)&:= x_c(t)\,,&\end{aligned}\right\} \quad t \in \mathbb {N}_0, \end{aligned}$$

demonstrating that (PI1) is an instance of (I), only with *A* replaced by $A-BF_1$. The same argument shows that (PI2) simplifies to (I) as well, now with *A* replaced by $A-BF_2C$. We do not give the details. The upshot is that Theorem 3.1 is applicable to (PI1) provided that $F_1$ can be chosen such that $A-BF_1$ satisfies (A1) and *K* can be chosen such that *K* and the transfer function of $(A_1,B,C)$ together satisfy (A2). In usual situations the crucial requirements is the choice of $F_1$ such that $r(A-BF_1) <1$, as here a suitable *K* in (PI1) is given by $K = (C(I -A_1)^{-1}B)^{-1}$ (see Remark 3.2 below). The analogous statements are true for (PI2).

Theorem 3.1 is the basis for the robust feedback control solution to the multiple management goals problem, motivated in Sect. 2. Additional features need to be included in the model (I) to cope with the demands of ecological management, and are done so in the next section. We conclude the current section by making some remarks on the roles of the dimensions of the input and output spaces, *m* and *p*, respectively, and also assumption (A2) that appears in the above theorem.

#### *Remark 3.2*

(i) In the case that $r(A)<1$, we see from (3.3) that the range of possible limiting outputs is equal to the image of *G*(1), which is at most *m*-dimensional. For every $r \in \mathbb {R}^p$ to belong to this image then necessarily we require that $m \ge p$ and that *G*(1) is surjective. In words, as many control actions are needed as observations are to be regulated. When $m > p$ then there is some redundancy, or non-uniqueness, in the choice of inputs.

(ii) For any $$m, p \in \mathbb {N}$$, assumption (A2) implies that *KG*(1) is invertible, as zero is not an eigenvalue of *KG*(1). In this case *G*(1) must be injective as if $$G(1)v = 0$$ for some $$v \in \mathbb {R}^m$$ then $$KG(1)v = 0$$ and thus $$v=0$$. Therefore, by the rank-nullity theorem, $$m \le p$$. In order for every reference $$r \in \mathbb {R}^p$$ to be a candidate limit of the output, we require that *G*(1) is surjective hence $$m \ge p$$ (as noted in (i)). Combined we see that necessarily $$m=p$$. Therefore, $$m =p $$ and (A2) together imply that *G*(1) is invertible, and hence the inverses in parts (a) and (b) of Theorem 3.1 make sense. Consequently, the spectrum condition (A2) implies that $$ K = KG(1)\cdot G(1)^{-1}$$ is invertible as well.
(iii) Conversely, if (as usual) $m = p$ and *G*(1) is invertible, then assumption (A2) is not restrictive. A candidate *K* is $G(1)^{-1}$ which clearly satisfies $\sigma (KG(1)) = \sigma (I) = \{1\}$ with positive real part. We note that $K = G(1)^{-1}$ requires knowledge of *G*(1). If *G*(1) is not known exactly then *K* can be based on an estimate of (the inverse of) *G*(1) which we investigate further in Sect. 4.4.3.

## Multi-input, multi-output low-gain PI control for positive systems with input saturation

Having recapped low-gain PI control for linear systems in Sect. 3 we now introduce additional structure that arises from considering positive state linear systems, our primary focus, and present a low-gain, multi-input, multi-output PI controller. Specifically, we additionally assume that (*A*, *B*, *C*) in (2.2) satisfy

4.1 $$\begin{aligned} (A,B,C) \in \mathbb {R}^{n \times n}_+ \times \mathbb {R}^{n \times m}_+ \times \mathbb {R}^{p \times n}_+, \end{aligned}$$

and all initial states $x^0$ are componentwise nonnegative, so that $x^0 \in \mathbb {R}^n_+$. As in Theorem 3.1, in our subsequent low-gain PI control results, we shall assume that $m=p$ (see Remark 3.2 for motivation of this choice).

The framework (2.2) and (4.1) includes matrix PPMs where the input, state and output of (2.2) denote the control action, the stage- or age-structured population abundances, and some measurement or observation of the population, respectively. In this applied context the assumption that $m=p$ means that as many per time-step measurements of the population are made as are available per-time step management actions.

We seek a version of Theorem 3.1 for asymptotic tracking of a chosen nonnegative set-point $r \in \mathbb {R}^m_+$. As motivated in Sect. 2, the two issues recorded there as (P1) and (P2) must be overcome. To that end, in Sect. 4.1 we describe the set of feasible set-points—these are candidate limits of the output of a positive state linear system where nonnegativity of the state and output variables is preserved (P1). Then, in Sect. 4.2, we establish stability of a low-gain integral control system with the additional feature that the input to the state equation is saturated (P2). Saturating the input introduces a nonlinearity into the feedback system, and that the conclusions of Theorem 3.1 still hold must be derived. Recall that the motivation for saturating the input is to avoid removing individuals when conservation is the ultimate goal and to reflect the realistic constraint of per-time step resource or capacity limits.

### Feasible set-points for positive state control systems

In this section we answer the question (P1): to which nonnegative set-points can the output *y* of (2.2) and (4.1) converge? Although we shall apply these results to inputs *u* generated by a PI controller, for now it suffices to consider convergent inputs. For that reason we do not need to impose the restriction $m=p$ in this section. We introduce some terminology and notation.

#### **Definition 4.1**

For (*A*, *B*, *C*) as in (3.1) we say that $r \in \mathbb {R}^p$ is *trackable* if there exists a convergent input *u* such that the output *y* of (2.2) converges to *r* as *t* tends to infinity. Supposing further that (*A*, *B*, *C*) satisfy (4.1) we say that $r \in \mathbb {R}^p_+$ is *trackable with positive state* if *r* is trackable and moreover the state *x*(*t*) of (2.2) is componentwise nonnegative for every $t \in \mathbb {N}_0$. We call the set of such *r* the set of trackable outputs of (*A*, *B*, *C*) with positive state.

We seek to characterise the set of trackable outputs of (*A*, *B*, *C*) with positive state. For $X \in \mathbb {R}^{s \times t}_+$, where $s,t \in \mathbb {N}$, the set $\langle X \rangle _+$ denotes all nonnegative linear combinations of the columns of *X*, which is a subset of $\mathbb {R}^{s}_+$. We also denote componentwise nonnegativity of a matrix *X* or vector *v* by $X \ge 0$ or $v \ge 0$ (respectively, also $0\le X$ and $0 \le v$).

We remind the reader that the subsequent claims are proved in “Appendix C”.

#### **Lemma 4.2**

Suppose that (*A*, *B*, *C*) is given by (4.1) and that $r(A) <1$. Then

- $$G_{CAB}(1) := C(I-A)^{-1}B \ge 0$$;
- for each $F \in \mathbb {R}^{n \times m}, F \ge 0$ such that $A_1 := A-BF \ge 0$, the set of trackable outputs of (*A*, *B*, *C*) with positive state contains $\langle G_{C A_1B}(1) \rangle _+$.

Next we recall an assumption from Guiver et al. (2014),^2^ which pertains to a nonnegative pair $(A,B) \in \mathbb {R}^{n \times n}_+ \times \mathbb {R}^{n \times m}_+$:

(H): There exists $F \in \mathbb {R}^{n \times m}, F \ge 0$ such that $A_1 := A-BF \ge 0$ and for any $v \in \mathbb {R}^n_+$ and $w \in \mathbb {R}^m$, if $A_1 v + Bw \ge 0$, then $w \ge 0$.

Assumption (H) for the pair (*A*, *B*) captures the situation whereby for any nonnegative *x* it is possible to choose negative *u* such that $Ax + Bu$ is “as small as possible”, yet still nonnegative. Indeed, the choice of *u* that achieves this is $u = -Fx$. Assumption (H) always holds if $B = b = e_i$, the $i{\mathrm{th}}$ standard basis vector, as then the required $F = f^T$ is the $i{\mathrm{th}}$ row of *A*. For instance, with

$$\begin{aligned} A = \begin{bmatrix} f_1&\quad f_2&\quad f_3 \\ g_1&\quad s_2&\quad 0 \\ 0&\quad g_2&\quad s_3 \end{bmatrix} \ge 0 \quad \text {and} \quad B = e_1 = \begin{bmatrix}1 \\ 0 \\0 \end{bmatrix}, \end{aligned}$$

then $$F = f^T = \begin{bmatrix}f_1&f_2&f_3 \end{bmatrix} \ge 0$$ gives

$$\begin{aligned} A_1 = A - bf^T = \begin{bmatrix}0&\quad 0&\quad 0 \\ g_1&\quad s_2&\quad 0 \\ 0&\quad g_2&\quad s_3 \end{bmatrix}\ge 0, \end{aligned}$$

and so if $A_1v + bw \ge 0$ then by inspection of the first component, necessarily $w \ge 0$. Assumption (H) always holds for any $A \in \mathbb {R}^{n \times n}_+$ when $B =[ c_{i_1} e_{i_1}, \ldots ,c_{i_k} e_{i_k}]$ for some distinct $i_j \in \{1,2,\dots , n\}$ with $c_{i_j} >0$ for each *j*. Furthermore, Guiver et al. (2014, Lemma 2.1) contains a constructive algorithm for checking whether assumption (H) holds for any pair (*A*, *B*), and determines the required *F* (which is unique) when it exists.

We have recalled assumption (H) because if the (*A*, *B*) component of (2.2) satisfies (H) then there exists a characterisation of the set of trackable outputs of (*A*, *B*, *C*) with positive state.

#### **Proposition 4.3**

Suppose that (*A*, *B*, *C*) is given by (4.1), $r(A) <1$ and additionally that the pair (*A*, *B*) satisfies assumption (H). Then the set of trackable outputs of (*A*, *B*, *C*) with positive state is precisely equal to

$$\begin{aligned} \langle G_{C A_1B}(1) \rangle _+ = \langle C(I - A_1)^{-1}B \rangle _+ , \end{aligned}$$

where $A_1$ is as in (H).

The next result provides a recipe for enlarging the guaranteed set of possible trackable outputs with positive state, particularly in the case that (H) is not satisfied.

#### **Lemma 4.4**

Suppose that (*A*, *B*, *C*) is given by (4.1) and that $r(A) < 1$. If $F \in \mathbb {R}^{n \times m}_+$ is such that $A_1 := A-BF \ge 0$ then

- $I - G_{F A_1 B}(1)$ is invertible, and;
- $$\langle G_{CAB}(1) \rangle _+ \subseteq \langle G_{C A_1B}(1) \rangle _+.$$

#### *Remark 4.5*

A straightforward adjustment to the proof of Lemma 4.4 demonstrates that the sets $\langle G_{C AB}(1) \rangle _+$ have a monotonically decreasing nested structure with respect to the partial ordering of componentwise nonnegativity on *A*, in that

$$\begin{aligned} 0 \le A \le {\bar{A}} \Rightarrow \langle G_{C {\bar{A}} B}(1) \rangle _+ \subseteq \langle G_{C A B}(1) \rangle _+, \end{aligned}$$

where $A \le {\bar{A}}$ means that $0\le {\bar{A}} - A$. The largest possible set that can be achieved by this process is $\langle CB \rangle _+$ and occurs when $F \ge 0$ can be chosen such that $A - BF = 0$. In this case the set of trackable outputs of (*A*, *B*, *C*) with positive state must contain $\langle CB \rangle _+$. Proposition 4.3 demonstrates that, when assumption (H) holds, $\langle G_{C A_1B}(1) \rangle _+$ is the largest possible set for tracking with positive state.

### Low-gain integral control with input saturation

In this section we address question (P2) by demonstrating that suitable adjustments to the integral control model (I), incorporating saturation on the input, achieve set-point regulation, as well as bounding the per time-step input and preserving nonnegativity of the state and output variables. Recall that the three-faceted motivation for saturating the input is to: (i) allow for the inclusion of per time-step bounds representing resource or capacity constraints associated with the implementation of a management strategy; (ii) prevent negative control signals particularly problematic when conservation is the desired outcome, and; (iii) prevent (meaningless) negative state and output variables. These three issues are all exhibited in Fig. 1 yet are resolved in Fig. 2.

**Fig. 3:**
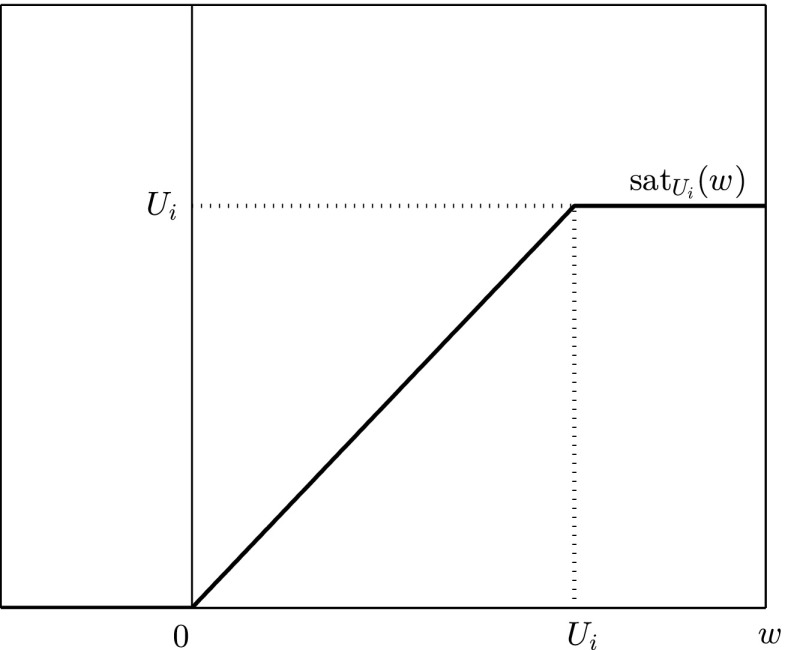
Graph of the saturation function $\mathrm{sat}_{U_i}$ defined in (4.2)

We next introduce the input saturation function which is incorporated into a low-gain integral control model in (Iaw). For given $U >0$ define the function $\mathrm{sat}\,_{U}$ by

4.2 $$\begin{aligned} \mathrm{sat}\,_{U} :\mathbb {R}\rightarrow \mathbb {R}, \quad w \mapsto \mathrm{sat}\,_{U}(w) := \left\{ \begin{array}{ll} 0, &{}\quad w<0 \\ w, &{}\quad 0\le w \le U, \\ U, &{}\quad U < w \end{array} \right. \end{aligned}$$

an example of which is graphed in Fig. 3. The diagonal saturation function $\mathrm{sat}\,$ is defined as the componentwise combination of $\mathrm{sat}_{U_i}$ functions as follows:

4.3 $$\begin{aligned} \mathrm{sat}\,:\mathbb {R}^m \rightarrow \mathbb {R}^m_+, \quad v \mapsto \mathrm{sat}\,(v) := [\mathrm{sat}\,_{U_1}(v_1) \mathrm{sat}\,_{U_2}(v_2) \ldots \mathrm{sat}\,_{U_m}(v_m)]^T. \end{aligned}$$

Here the constants $U_i>0$ for $i \in \{1,2,\dots ,m\}$ are chosen and in applications denote the per time-step bound on the $i{\mathrm{th}}$ component of the input. To incorporate input saturation into a low-gain integral control model we consider:

Iaw $$\begin{aligned} \left. \begin{aligned} x(t+1)&= Ax(t) + Bu(t),&\quad x(0) \!=\! x^0, \\ x_c(t+1)&= x_c(t) \!+\! gK(r- Cx(t)) \!-\! E(x_c(t) \!-\! \mathrm{sat}\,(x_c(t))),&\quad x_c(0) \!=\! x_c^0, \\ u(t)&= \mathrm{sat}\,(x_c(t)),&\end{aligned} \right\} \, t \in \mathbb {N}_0, \end{aligned}$$

where $E \in \mathbb {R}^{m \times m}$ is a design parameter additional to those appearing in (I) and is discussed in more detail in Sect. 4.3. Our main result of the manuscript is Theorem 4.6 below, which mirrors Theorem 3.1, and guarantees that the low-gain integral control model with input saturation (Iaw) achieves asymptotic tracking of the output of (Iaw) to a prescribed set-point under the (same, previously employed) assumptions (A1) and (A2) and a known choice of *E*. The theorem provides solutions to problems (P1) and (P2).

#### **Theorem 4.6**

Suppose that (Iaw) satisfies (A1) and (A2) and choose

4.4 $$\begin{aligned} E := gKG(1), \end{aligned}$$

where $g >0$ is as in (Iaw). Then, there exists $g^*>0$ such that for all $g \in (0,g^*)$, all $r \in \langle G(1)\rangle _+$ such that

4.5 $$\begin{aligned} r = G(1)u_+, \quad \text {for some }u_+ \in \mathbb {R}^m_+ \ \text {with} \ u_+ \le U, \end{aligned}$$

and all $(x^0, x^0_c) \in \mathbb {R}^n_+ \times \mathbb {R}^m_+$, the solution $(x,x_c)$ of (Iaw) satisfies $x(t) \ge 0$ for each $t \in \mathbb {N}_0$ and has the properties:

- $$\displaystyle {\lim \nolimits _{t\rightarrow \infty }}x_c(t)= x_c^\infty : = G(1)^{-1}r$$;
- $$\displaystyle {\lim \nolimits _{t\rightarrow \infty }}x(t)= x^\infty := (I-A)^{-1}BG(1)^{-1}r$$;
- $$\displaystyle {\lim \nolimits _{t\rightarrow \infty }}y(t)= \displaystyle {\lim \nolimits _{t\rightarrow \infty }}Cx(t)= r$$.

By appealing to the results of Sect. 4.1, including a proportional component in the feedback law in (Iaw) gives rise to a larger set of candidate set-points. Let (PI1aw) denote the feedback system

PI1aw $$\begin{aligned} \left. \begin{aligned} x(t+1)&= Ax(t) + Bu(t),&\quad x(0) = x^0, \\ x_c(t\!+\!1)&= x_c(t) \!+\! gK(r\!-\! Cx(t)) \!-\! E(x_c(t)\! -\! \mathrm{sat}\,(x_c(t))),&\, x_c(0) \!=\! x_c^0, \\ u(t)&= -F_1 x(t) +\mathrm{sat}\,(x_c(t)),&\end{aligned} \right\} \quad \!\! t \!\in \! \mathbb {N}_0, \end{aligned}$$

which differs from (Iaw) only by the inclusion of an additional proportional state-feedback $-F_1x(t)$ to the updated input $u = -F_1 x +\mathrm{sat}\,(x_c)$. As before, $F_1 \in \mathbb {R}^{m \times n}$ is another design parameter. We let (PI2aw) denote the feedback system (PI1aw) with $-F_1x(t)$ instead replaced by $-F_2y(t) = -F_2Cx(t)$, that is, an output-feedback which replaces *u* in (PI1aw) $u = -F_2 C x +\mathrm{sat}\,(x_c)$. Again, $F_2 \in \mathbb {R}^{m \times m}$ is a design parameter. We present the following corollary for the low-gain PI control systems (PI1aw) and (PI2aw).

#### **Corollary 4.7**

The low-gain PI systems (PI1aw) and (PI2aw) specified by (*A*, *B*, *C*) satisfying (4.1) are equal to (Iaw) specified by $$(A_1,B,C)$$ and $$(A_2,B,C)$$, where $$ A_1 := A - BF_1$$ and $$ A_2 := A - BF_2C$$, respectively. If $$F_1$$ is such that $$A_1 \ge 0$$ and $$(A_1,B,C)$$ satisfy (A1) and (A2) then the conclusions of Theorem 4.6 apply to (Iaw) specified by $$(A_1,B,C)$$, and similarly for $$F_2$$.

### Comparing and contrasting low-gain feedback systems (I) and (Iaw)

In this section we record some observations on the low-gain integral control system (Iaw), the above theorem and corollary, and their relation to other published results. The integral control scheme (Iaw) differs from (I) by the saturation function in the definition of *u*, and by the term involving *E* appended to controller state dynamics as well. The term involving *E* is crucial and, intuitively, acts as a correction term, activating at time-steps *t* when the integral control state $x_c(t)$ saturates meaning that $x_c(t) \ne \mathrm{sat}\,(x_c(t))$. The input is *not* saturated when $\mathrm{sat}\,(x_c(t)) = x_c(t)$ and for these time-steps the term in (Iaw) involving *E* is zero and plays no role. Loosely speaking, at these times (Iaw) is behaving as though there is no saturation, the resulting model is linear and Theorem 3.1 applies. Theorem 4.6 makes the previous assertion rigorous.

The feedback system (Iaw) with $E =0$ was considered in Guiver et al. (2015) in the specific so-called single-input, single-output case (meaning $m = p = 1$), so that $B = b$ and $C = c^T$ are vectors. Here assumption (A1) is as before, and assumption (A2) reduces to $G(1) > 0$ (it suffices to take $K = 1$). However, in contrast to the situation in Guiver et al. (2015), saturating a multi-input ($m > 1$) control signal can be inherently *destabilising*, resulting in the desired set-point regulation objective *not* being achieved. Roughly, if $E=0$ then the control signal may get ‘stuck’ in the saturating region, and the resulting failure is attributed to what is known as “actuator saturation” or “integrator windup” in control engineering literature (Johanastrom and Rundqwist 1989). Anti-windup control refers to the study of mechanisms to alleviate or remove windup in PI controllers and, owing to its importance in applications, is a hugely well-studied topic. The already 20-year-old chronological bibliography of Bernstein and Michel (1995) contains 250 references. We refer the reader to Tarbouriech and Turner (2009) for a recent overview of anti-windup control. There are many possible such mechanisms, for example in how to choose the matrix *E* that appears in (Iaw), also known as a static anti-windup component. The advantages of our choice of *E* in Theorem 4.6 and elsewhere are that it:

(i) is straightforward to compute and thus implement;
(ii) possesses demonstrable robustness to model uncertainty, and;
(iii) can be extended to a class of infinite-dimensional systems.

For readers less familiar with (or indeed interested in) anti-windup control, the key feature of the present discussion is that the term involving *E* in (Iaw) is a crucial feature and should not be omitted. We reiterate that although our results are aimed at ecological models, they apply to any positive state linear system described by (2.2). As far as we know, the anti-windup method we propose and its proof is novel in a control theory context as well.

One approach to anti-windup control present in the literature determines the anti-windup component *E* via the solution of a set of certain linear matrix inequalities (LMIs) see, for example, Mulder et al. (2001) or Silva and Tarbouriech (2006). Although these LMIs can often be solved numerically and can result in other performance criteria being met (such as so-called “bumpless transfer”), they introduce another level of complexity for the modeller. Moreover, since they use Lyapunov based arguments, they seemingly do not extend across to systems that have infinite-dimensional Banach spaces as state-spaces (thus precluding IPMs, for instance).

#### *Remark 4.8*

(i) Although when $r(A) <1$ no $F_1$ or $F_2$ component is required to apply Theorem 4.6, the use of (PI1aw) or (PI2aw) often results in faster convergence than that of (Iaw), highlighted as issue (iv) in Sect. 2. Moreover, if $F_1 \in \mathbb {R}^{n \times n}$ is such that
  $$\begin{aligned} 0 \le A- BF_1 \le A, \end{aligned}$$
  then Lemma 4.4 (b) implies that there is a larger choice of possible references achievable by (PI1aw) than by (Iaw), which thus encourages the use of PI control even in the case that $r(A) <1$. Similar comments apply to $F_2$ for the (PI2aw) system.
(ii) If *K* and *G*(1) are such that *KG*(1) is positive semi-definite then it can be shown that the conclusions of Theorem 4.6 hold for (Iaw) with $E = 0$, that is, with no anti-windup component. Although the choice $K = G(1)^{-1}$ guarantees this condition, such a choice requires exact knowledge of *G*(1) and the requirement that *KG*(1) is positive semi-definite is very non-robust to parameter uncertainty. For this reason we have, therefore, insisted on including the anti-windup component $E(x_c - \mathrm{sat}\,(x_c))$ in (Iaw).
(iii) As mentioned in the introduction, feedback control that preserves nonnegativity of state and solves a non-zero state-regulation problem has been considered in Nersesov et al. (2004). The goals of that paper and ours here are similar, but there the authors work in continuous-time, and use a feedback derived from a constrained optimal control problem (as opposed to a low-gain integral controller) to steer the state to a prescribed non-zero equilibrium. They do not consider input saturation to avoid negative states but instead constrain the structure of the inputs. Their work builds on that of Leenheer and Aeyels (2001). Roszak and Davison (2009) solve the continuous-time, nonnegative output regulation problem (also called the servomechanism problem, hence their title) using low-gain integral control. There, the authors determine the model parameter *K* in (I) using optimal control results—a different approach to ours. Another key difference between that work and ours is that the input is not saturated (that is, bounded) from above and thus, as we understand, “integrator windup” is not an issue.

### Robustness of low-gain PI control

The efficacy of the low-gain PI control systems considered so far is predicated on several modelling assumptions:

1. that the system of interest is accurately modelled by (2.2) and (4.1);
2. there are no external signals or noises affecting the dynamics of the state *x* or the input *u*;
3. there is no measurement or sampling error in *y*;
4. the steady-state gain *G*(1) is known.

In practice, all four of these assumptions are likely to be violated and thus here we quantify to what extent low-gain PI control is robust to failures of (U1)–(U4). By doing so we seek to describe how concepts from robust feedback control apply to sources of uncertainty that arise in ecological modelling. A more detailed discussion may be found in Guiver et al. (2015, Section 3.1), but briefly, Table 1 [based on Guiver et al. (2015, Table 1)] connects the list above with sources of uncertainty described in the ecology literature.

**Table 1:** Connecting sources of uncertainty present in low-gain PI control with uncertainty terminology appearing in the ecology literature

	Williams (2001)	Regan et al. (2002)
(U1)	Structural uncertainty	Natural variation
		Model uncertainty
(U2)	Environmental variation	Inherent randomness
	Partial controllability	
(U3)	Partial observability	Measurement error
		Systematic error
(U4)	Structural uncertainty	Model uncertainty

#### Robustness to choice of model structure and model parameters

When modelling ecological processes, such as managed populations, there are often a plethora of models to choose from that all attempt to capture the same underlying dynamics. Within structured population models of the form (2.1) there are age- or size- based models, that partition the life cycle (perhaps a continuum of stages) into predetermined discrete stage-classes. The above choices imply that there is choice, or indeed, uncertainty in *A*, *B* and *C* in (2.1), challenging (U1). The state dimension *n* may even be uncertain. Low-gain PI control is robust to this source of uncertainty in the sense that knowledge of *A*, *B* and *C* is not required to implement it. The measured variable *y*(*t*) is required, and it is assumed that $y(t) = C x(t)$, for *some* choice of *C*, but *C* itself is not needed. Rather, *A*, *B* and *C* are required to satisfy the assumptions (A1) and (A2). Assumption (A1) does not need *A* to be known, and simply means that the population of interest is in decline. Recall that when seeking to use PI control to reduce a growing population, then assumption (A1) amounts to the requirement that the population can be stabilised (that is, made to decline) by state- or output-feedback—see (PI1) and the discussion below. Assumption (A2) does require knowledge of *G*(1) to determine a suitable *K* (and *E* for (Iaw)), which may be determined from *A*, *B* and *C*, but may also be known by experiment or experience. As we explain in Sect. 4.4.3, an estimate of *G*(1) maybe sufficient to determine a *K* that together satisfy (A2). Finally, we comment that since assumptions (A1) and (A2) are necessary for low-gain integral control (as well as sufficient), we cannot allow any greater model uncertainty.

#### Robustness to external disturbances

External dynamics affecting the state, input and output may be included in the original model (2.2) by writing:

4.6 $$\begin{aligned} \left. \begin{aligned} x(t+1)&= Ax(t) + Bu(t) + d_1(t),&\quad x(0) = x^0, \\ y(t)&= Cx(t) + d_2(t),&\end{aligned}\right\} \quad t \in \mathbb {N}_0, \end{aligned}$$

where $d_1(t) \in \mathbb {R}^n$ and $d_2(t) \in \mathbb {R}^p$ are typically unknown. In a population model $d_1$ may denote either a disturbance to the population such as (unmodelled) immigration, emigration or predation or an input error, meaning that the intended input *u*(*t*) is disturbed. Similarly, $d_2$ denotes some form of measurement or sampling error. The inclusion of $d_1$ and $d_2$ seeks to address the assumptions (U2) and (U3). A reasonably general framework is to assume that $d_1$ and $d_2$ in (4.6) are bounded, and of course are such that *x* and *y* remain nonnegative. We refer the reader to Eager et al. (2014) and the references therein for more information on the impacts of nonnegative disturbances on populations modelled by matrix PPMs.

When only boundedness of $d_1$ and $d_2$ is assumed then we cannot in general expect the same convergence of the output *y* of the feedback system (4.6) connected with a low-gain PI controller as that exhibited by (Iaw), (PI1aw) or (PI2aw). The next result provides upper bounds on the difference of the state and output from their respective asymptotic limits in terms of the initial error and the maximum values of $d_1$ and $d_2$. The result is an (I)nput-to-(S)tate-(S)tability estimate and we refer the reader to Sontag (2008) for more background on ISS.

##### **Proposition 4.9**

Suppose that the low-gain integral control system with disturbances

4.7 $$\begin{aligned} \left. \begin{aligned} x(t+1)&= Ax(t) + Bu(t) + d_1(t),&x(0) = x^0, \\ y(t)&= Cx(t) + d_2(t),&\\ x_c(t+1)&= x_c(t) \!+\! gK(r- y(t))\! -\! E(x_c(t) \!-\! \mathrm{sat}\,(x_c(t))),&x_c(0) \!=\! x_c^0, \\ u(t)&= \mathrm{sat}\,(x_c(t)),&\end{aligned}\right\} \, t \in \mathbb {N}_0,\nonumber \\ \end{aligned}$$

with bounded disturbances $d_1$ and $d_2$ satisfies (A1) and (A2), and choose $E := gKG(1)$ where $g >0$ is as in (Iaw). Then, there exists $g^*>0$ such that for all $g \in (0,g^*)$, all *r* as in (4.5) and all $(x^0, x^0_c) \in \mathbb {R}^n_+ \times \mathbb {R}^m_+$, the solution $(x,x_c)$ of (4.7) satisfies

4.8 $$\begin{aligned} \left\| \begin{bmatrix}x(t) - x^\infty \\ x_c(t) - x_c^\infty \\ y(t)-r \end{bmatrix} \right\|\le & {} M_0 \gamma ^t \left\| \begin{bmatrix} x^0 - x^\infty \\ x_c^0 - x_c^\infty \end{bmatrix} \right\| + M_1 \max _{ \mathop {j \in \mathbb {N}_0 }\limits _{j \le t-1}}\Vert d_1(j) \Vert \nonumber \\&+ M_2 \max _{ \mathop {j \in \mathbb {N}_0 }\limits _{j \le t-1}} \Vert d_2(j) \Vert , \quad t \in \mathbb {N}, \end{aligned}$$

for some constant $\gamma \in (0,1)$ and $M_0,M_1,M_2>0$ and where $x^\infty _c$ and $x^\infty$ are as in (a) and (b) of Theorem 3.1, respectively. The constants $\gamma , M_0,M_1$ and $M_2$ depend on *A*, *B*, *C*, *g* and *K*, but not on *r*, $x^0$, $x_c^0$, $d_1$ or $d_2$. Furthermore, $g^*$ is independent of the disturbances $d_1$ and $d_2$.

Low-gain PI control without saturation or positivity constraints is known to have the desirable property that convergent input disturbances $d_1 = B f_1$, for some disturbance $f_1$, are rejected by the integral controller, meaning that the output still converges to the desired set-point. Meanwhile, convergent output disturbances $d_2$ result in asymptotic tracking of the output to the set-point offset by the limit of the disturbance. A convergent output disturbance includes constant disturbances which may, for example, correspond to a systematic or persistent measurement error. The next corollary demonstrates that, broadly speaking, the same disturbance rejection and offset in the set-point properties hold for the low-gain integral control model (Iaw) with input saturation.

##### **Corollary 4.10**

Suppose that the low-gain integral control system with disturbances (4.7) satisfies (A1) and (A2), and choose $E := gKG(1)$ where $g >0$ is as in (Iaw). Suppose that $f_1$ and $d_2$ are convergent with respective limits $f_1^\infty$ and $d_2^\infty$. Then, there exists $g^*>0$ such that for all $g \in (0,g^*)$, all $r \in \mathbb {R}^m_+$ such that

4.9 $$\begin{aligned} r - d_2^\infty = G(1)(u_+ + f_1^\infty ), \quad \text {for some }u_+ \in \mathbb {R}^m_+\hbox { such that }u_+ \le U, \end{aligned}$$

and all $(x^0, x^0_c) \in \mathbb {R}^n_+ \times \mathbb {R}^m_+$, the solution $(x,x_c)$ of (4.7) satisfies:

- $$\displaystyle {\lim \nolimits _{t\rightarrow \infty }}x_c(t) = G(1)^{-1}(r-d_2^\infty ) = u_+ + f_1^\infty $$,
- $$\displaystyle {\lim \nolimits _{t\rightarrow \infty }}x(t) = (I-A)^{-1}BG(1)^{-1}(r-d_2^\infty ) = (I-A)^{-1}B(u_+ + f_1^\infty )$$,
- $$\displaystyle {\lim \nolimits _{t\rightarrow \infty }}y(t) = \displaystyle {\lim \nolimits _{t\rightarrow \infty }}Cx(t)= r-d_2^\infty $$.

The constant $g^*$ is independent of $f_1$ and $d_2$.

#### Robustness to uncertainty in the steady-state gain *G*(1)

In the final part of our material on robustness with respect to various forms of uncertainty, here we consider the situation where the steady-state gain matrix *G*(1) is not known precisely, meaning that (U1) is violated. Knowledge of *G*(1) is used in low-gain integral control and PI control in three separate situations: first, in determining *K* to satisfy (A2) that appears in the original integral control model (I), the PI models (PI1), (PI2) and, the focus of the present study, (Iaw). Second, *G*(1) is used in determining *E* that appears in (Iaw). Both of the components *K* and *E* are required to ensure that low-gain PI control as presented is effective as described in Sect. 4.3. Recall that the choice $K = G(1)^{-1}$ satisfies (A2) and $E = gKG(1)$ is sufficient to ensure that the conclusions of Theorem 4.6 (and Corollaries 4.7, 4.10, Proposition 4.9) hold. Third, knowledge of *G*(1) is used in Lemma 4.2 to help determine the set of trackable outputs with positive state, which in turn provides feasible set-points.

Uncertainty in *G*(1) typically arises from uncertainty in the parameters or even the dimensions of *A*, *B* or *C*. Throughout this section we shall assume that the unknown transfer function *G* in (3.2) can be decomposed as

4.10 $$\begin{aligned} G = {\hat{G}} + {\Delta } G, \end{aligned}$$

where ${\hat{G}}$ is known and ${\Delta } G$ is expected to be “small”. More generally, in this section variables with hats shall always denote known quantities and capital deltas denote uncertain terms.

Lemmas 4.11–4.13 below are technical preliminary results gathering sufficient conditions for the main result of the section, Corollary 4.15. This latter result states that if a known nominal estimate ${\hat{G}}$ is close to the unknown *G*, meaning that $\Vert G - {\hat{G}}\Vert _\infty$ is small^3^, then basing the design of *K* and *E* on the nominal estimate ${\hat{G}}(1)$ of *G*(1) is sufficient for low-gain PI control to succeed.

We first demonstrate how the decomposition (4.10) arises from parametric uncertainty in *A*, *B* and *C*. We let $\rho (A) = \mathbb {C}{\setminus } \sigma (A)$ denote the resolvent set of *A*, (when *A* is a matrix then $\rho (A)$ is the set of all complex numbers that are *not* eigenvalues of *A*).

##### **Lemma 4.11**

Suppose that $(A,B,C) \in \mathbb {R}^{n \times n} \times \mathbb {R}^{n \times m} \times \mathbb {R}^{m \times n}$ for $m,n \in \mathbb {N}$ admit the decompositions

$$\begin{aligned} A = {\hat{A}} + {\Delta } A, \quad B = {\hat{B}} + {\Delta } B, \quad C = {\hat{C}} + {\Delta } C, \end{aligned}$$

then

4.11 $$\begin{aligned} G_{CAB}(z)&= C(zI -A)^{-1}B = G_{{\hat{C}} {\hat{A}} {\hat{B}}}(z) + {\Delta } C(zI - {\hat{A}})^{-1}({\hat{B}} + {\Delta } B)\nonumber \\&\quad + {\hat{C}} (zI - {\hat{A}})^{-1} {\Delta } B + ({\hat{C}} + {\Delta } C)(zI - A)^{-1} {\Delta }A (zI - {\hat{A}})^{-1}( {\hat{B}}+{\Delta } B) , \end{aligned}$$

which is defined for all $z \in \mathbb {C}\cap \rho (A) \cap \rho ({\hat{A}})$ and is in the form (4.10) with ${\hat{G}} = G_{{\hat{C}} {\hat{A}} {\hat{B}}}$ and ${\Delta } G$ the sum of the remaining three terms on the right hand side of (4.11).

##### **Lemma 4.12**

Suppose that *G* admits the decomposition (4.10) and that ${\hat{G}}(1)$ is invertible. Choose $K = Q \hat{G}(1)^{-1}$, where $Q \in \mathbb {C}^{m \times m}$ is such that $\sigma (Q) \subseteq \mathbb {C}_0^+$. Then assumption (A2) is satisfied for *K* and *G*(1) if

4.12 $$\begin{aligned} \Vert Q {\hat{G}}(1)^{-1} {\Delta } G(1) \Vert < \frac{1}{\sup _{\omega \in \mathbb {R}}\Vert (\omega \mathrm {i}+ Q)^{-1} \Vert }. \end{aligned}$$

If $Q =I$, the $m \times m$ identity matrix, then *K* and *G*(1) satisfy (A2) if

4.13 $$\begin{aligned} \min \{ \Vert {\Delta } G(1) {\hat{G}}(1)^{-1} \Vert , \Vert {\hat{G}}(1)^{-1} {\Delta } G(1) \Vert \} < 1. \end{aligned}$$

A sufficient condition for (4.13) is that

4.14 $$\begin{aligned} \Vert {\Delta } G(1) \Vert < \frac{1}{\Vert {\hat{G}}(1)^{-1} \Vert }. \end{aligned}$$

##### **Lemma 4.13**

Let *X* denote a bounded operator on a Hilbert space (such as a square matrix with real or complex entries), with $-1 \in \rho (X)$, so that $I+X$ is invertible. Then the conditions

- $$ \Vert X \Vert < \tfrac{1}{2}$$, or;
- $$\Vert X \Vert \le 1$$ and $$\Vert (I-X)(I+X)^{-1} \Vert \le 1$$;

are sufficient for

4.15 $$\begin{aligned} \Vert (I+X)^{-1} X \Vert <1. \end{aligned}$$

##### *Remark 4.14*

An estimate of the form (4.15) appears as a condition on *X* in Corollary 4.15 below, hence the inclusion of sufficient conditions here. We comment that (a) and (b) do not imply one another as $X = -\tfrac{1}{4}I$ satisfies (a) but not (b) and $X = I$ satisfies (b) but not (a).

##### **Corollary 4.15**

Suppose that (*A*, *B*, *C*) as in (4.1) satisfy (A1) and the associated transfer function *G* admits the decomposition (4.10), where ${\hat{G}}$ is known and *K* and ${\hat{G}}$ together satisfy (A2) and choose

4.16 $$\begin{aligned} E := gK{\hat{G}}(1), \end{aligned}$$

where $g >0$ is as in (Iaw). Then, there exists $M^*>0$ and $g^*>0$ (which in general depends on $M^*$) such that for all ${\Delta } G$ in (4.10) with

4.17 $$\begin{aligned} \Vert {\Delta } G \Vert _\infty < M^*, \end{aligned}$$

and

4.18 $$\begin{aligned} \Vert [I + {\hat{G}}(1)^{-1} {\Delta } G(1) ]^{-1} \cdot [{\hat{G}}(1)^{-1} {\Delta } G(1)] \Vert <1, \end{aligned}$$

all $g \in (0,g^*)$, all $r \in \langle G(1)\rangle _+$ as in (4.5) and all $(x^0, x^0_c) \in \mathbb {R}^n_+ \times \mathbb {R}^m_+$, the solution $(x,x_c)$ of (Iaw) satisfies $x(t) \ge 0$ for each $t\in \mathbb {N}_0$ and has the properties (a), ( b) and (c) of Theorem 3.1.

##### *Remark 4.16*

Corollary 4.15 can easily be extended to the PI systems (PI1aw) or (PI2aw), considered in Corollary 4.7, by replacing *A* by $A_1$ and $A_2$ as appropriate.

### Low-gain PI control with input saturation for a class of infinite-dimensional systems

We have so far focussed on solving the robust set-point regulation problem with multiple management goals by applying low-gain PI control in the situation where the underlying (ecological) model is assumed finite-dimensional. Abstractly, we have developed low-gain PI control with input saturation for discrete-time positive-state linear systems. In this section we demonstrate that many of the results presented extend to a class of discrete-time, infinite-dimensional linear systems which includes the class of IPMs. IPMs were introduced by Easterling et al. (2000) (see also Ellner and Rees 2006; Rees and Ellner 2009 or Briggs et al. 2010) as a tool for population modelling where the *n* discrete age-, size- or stage-classes of a PPM are replaced by a continuous variable. As a concrete example, a shrub or tree population model may partition individuals according to a continuous variable denoting height or stem diameter. An IPM is a discrete-time linear system on the function space $L^1({\varOmega })$ specified by integral operator:

4.19 $$\begin{aligned} A :L^1({\varOmega }) \rightarrow L^1({\varOmega }), \quad (A v)(\xi )= & {} \int _{\varOmega } k(\zeta ,\xi ) v(\zeta ) \, d\zeta , \quad v \in L^1({\varOmega }), \nonumber \\&\text {almost all }\xi \in {\varOmega }, \end{aligned}$$

for some nonnegative-valued kernel

4.20 $$\begin{aligned} {\varOmega } \times {\varOmega } \ni (\zeta ,\xi ) \mapsto k(\zeta ,\xi ) \ge 0, \end{aligned}$$

where, for simplicity say, ${\varOmega }$ is the closure of some bounded set in $\mathbb {R}^n$, $n \in \mathbb {N}$. At each time-step $t \in \mathbb {N}_0$, the state of an IPM is a function of the continuous variable $\xi \in {\varOmega }$.

To formulate integral control in a possibly infinite-dimensional setting let $\mathcal {X}$ denote an ordered real Banach space, so that $\mathcal {X}$ is equipped with a partial order $\le$ (also $\ge$) that respects vector space addition and multiplication by nonnegative scalars. The positive cone $\mathcal {C}$ induced by $(\mathcal {B},\ge )$ is the set of $x \in \mathcal {X}$ such that $x \ge 0$ and is a closed, convex set (so that if $x, y \in \mathcal {C}$ and $\alpha \ge 0$ then $x+y, \alpha x \in \mathcal {C}$) with the property that $x, -x \in \mathcal {C}$ implies that $x =0$. For real Banach spaces $\mathcal {X}_1, \mathcal {X}_2$ with respective positive cones $\mathcal {C}_1, \mathcal {C}_2$ a bounded linear operator $T:\mathcal {X}_1 \rightarrow \mathcal {X}_2$ is called positive if $T \mathcal {C}_1 \subseteq \mathcal {C}_2$. In words, *T* is positive if every positive element of $\mathcal {X}_1$ is mapped to a positive element of $\mathcal {X}_2$.

#### *Example 4.17*

(i) The situation considered throughout the manuscript thus far has taken $\mathcal {X}= \mathbb {R}^n$ for $n \in \mathbb {N}$ with partial order $\ge$ denoting usual componentwise nonnegativity, so that $x \in \mathbb {R}^n$, $x \ge 0$ if $x_k \ge 0$ for every $k \in \{1,2,\dots ,n\}$. As such, the positive cone of $\mathbb {R}^n$ and this partial ordering is the nonnegative orthant $\mathcal {C}=\mathbb {R}^n_+$.
(ii) To model IPMs we choose $\mathcal {X}= L^1({\varOmega })$ with the partial ordering $\ge$ of almost everywhere pointwise inequality, that is $f \in L^1({\varOmega })$, $f \ge 0$ if $f(\xi ) \ge 0$ for almost all $\xi \in {\varOmega }$. With this choice of partial ordering the nonnegativity assumption in (4.20) implies that *A* in (4.19) is a positive operator. Moreover, by Krasnosel’skij et al. (1989, Theorem 2.1), Eq. (4.20) is sufficient to infer that *A* in (4.19) is a bounded operator. $\square$

Consider the linear system (2.2) where now

4.21 $$\begin{aligned} A :\mathcal {X}\rightarrow \mathcal {X}, \quad B :\mathbb {R}^m \rightarrow \mathcal {X}, \quad C:\mathcal {X}\rightarrow \mathbb {R}^m, \end{aligned}$$

are bounded, positive, linear operators and $\mathcal {X}$ is as above. The state-space $\mathcal {X}$ may now be infinite-dimensional but, for simplicity, the input and output spaces are still assumed to be $\mathbb {R}^m$. Since *B* and *C* are bounded and finite-rank, then necessarily they can be written

4.22 $$\begin{aligned} Bu&= \sum _{i =1}^m b_i u_i, \quad \forall u = \begin{bmatrix}u_1&\dots&u_m \end{bmatrix}^T \in \mathbb {R}^m, \end{aligned}$$

4.23 $$\begin{aligned} \text {and} \quad (C x)_j&= c_j x, \quad \forall j \in \{1,2,\dots ,m\}, \; x \in \mathcal {X}, \end{aligned}$$

for some $b_i \in \mathcal {X}$ and $c_j : \mathcal {X}\rightarrow \mathbb {R}$, linear functionals on $\mathcal {X}$. Using the expression (4.22), *B* is positive if, and only if, $b_i \in \mathcal {C}$ for every $i \in \{1,2,\dots , m\}$. Similarly, *C* is positive if, and only if, $c_j$ is positive for every $j \in \{1,2,\dots , m\}$, here meaning that $c_j(\mathcal {C}) \subseteq \mathbb {R}_+$.

The low-gain integral control system with input saturation is still defined by (Iaw), with design parameters $E,K \in \mathbb {R}^{m \times m}$, $g >0$ and $x_c^0 \in \mathbb {R}^m$. Note that for each time-step $t \in \mathbb {N}_0$, the integral controller state $x_c(t) \in \mathbb {R}^m$ is still finite-dimensional and thus readily computable. The expression (3.2) for the transfer function *G* is well-defined when *A*, *B* and *C* are as in (4.21), and consequently assumptions (A1) and (A2) are as before.

To include a (P)roportional feedback in the control law as in (PI1aw) or (PI2aw) requires bounded linear operators

4.24 $$\begin{aligned} F_1 :\mathcal {X}\rightarrow \mathcal {X}\quad \text {or} \quad F_2 :\mathbb {R}^m \rightarrow \mathcal {X}, \end{aligned}$$

respectively. When $F_1$ and $F_2$ are bounded then so are

4.25 $$\begin{aligned} A_1, A_2 :\mathcal {X}\rightarrow \mathcal {X}, \quad A_1 := A - BF_1, \quad A_2 := A - BF_2 C, \end{aligned}$$

as the composition and difference of bounded operators.

The main result of this section demonstrates that the low-gain integral controller (Iaw) still achieves the robust set-point regulation problem in the more general case when (*A*, *B*, *C*) are as in (4.21). By noting that the PI system (PI1aw) with $F_1$ or $F_2$ as in (4.24) reduces to (Iaw) with *A* replaced by $A_1$ or $A_2$ given by (4.25), the next result includes both the state- and output-feedback cases.

#### **Theorem 4.18**

Assume that the low-gain integral control feedback system (Iaw) specified by positive operators (*A*, *B*, *C*) in (4.21) satisfies assumptions (A1) and (A2) with $E = gKG(1)$ in (Iaw). Then, there exists $g^*>0$ such that for all $g \in (0,g^*)$, all *r* as in (4.5) and all $(x^0, x^0_c) \in \mathcal {C}\times \mathbb {R}^m_+$, the solution $(x,x_c)$ of (Iaw) has the properties (a), (b) and (c) of Theorem 3.1 and furthermore $x(t) \in \mathcal {C}$ for every $t \in \mathbb {N}_0$.

The robustness results Proposition 4.9, Corollary 4.10 and Corollary 4.15 also apply when (Iaw) is specified by positive operators (*A*, *B*, *C*) in (4.21).

The proofs of the above results are exactly the same as the earlier named results; none of the arguments used there required that $\mathcal {X}$ is finite-dimensional.

#### *Remark 4.19*

The results of Sect. 4.1 on feasible nonnegative set-points translate to the situation when $\mathcal {X}$ is a real, partially ordered Banach space. Again, none of the proofs explicitly use that $\mathcal {X}$ is finite-dimensional. However, assumption (H) should be replaced by:

$$({\mathrm{H}}^{\prime })$$
Let $\mathcal {X}$ denote a real, partially ordered Banach space with positive cone $\mathcal {C}$. Given the pair of bounded, linear, positive operators $A:\mathcal {X}\rightarrow \mathcal {X}$, $B:\mathbb {R}^m \rightarrow \mathcal {X}$ there exists a bounded, positive operator $F :\mathcal {X}\rightarrow \mathbb {R}^m$ such that defining ${\hat{A}} := A-BF$ it follows that ${\hat{A}}$ is positive and for any $v \in \mathcal {C}$ and $w \in \mathbb {R}^m$, if ${\hat{A}} v + Bw \in \mathcal {C}$ then $w \in \mathbb {R}_+^m$.

Importantly, the constructive characterisation Guiver et al. (2014, Lemma 2.1) *does not* hold in the general Banach space case, however, as it is truly a finite-dimensional result.

## Examples

### *Example 5.1*

Matrix projection models for the sustainable harvesting of two species of palm trees in Mexico are considered in Olmsted and Alvarez-Buylla (1995). We use a matrix PPM from there of the palm species *Coccothrinax readii* to demonstrate how a potential harvesting and conservation strategy could be based on a low-gain PI control law. The projection matrix *A* is given by:

5.1 $$\begin{aligned} A = \begin{bmatrix}0.35&\quad 0&\quad 0&\quad 0&\quad 0&\quad 0&\quad 0&\quad 0&\quad 55.8\\ 0.18&\quad 0.8&\quad 0&\quad 0&\quad 0&\quad 0&\quad 0&\quad 0&\quad 0\\ 0&\quad 0.1&\quad 0.89&\quad 0&\quad 0&\quad 0&\quad 0&\quad 0&\quad 0\\ 0&\quad 0&\quad 0.07&\quad 0.94&\quad 0&\quad 0&\quad 0&\quad 0&\quad 0\\ 0&\quad 0&\quad 0&\quad 0.06&\quad 0.92&\quad 0&\quad 0&\quad 0&\quad 0\\ 0&\quad 0&\quad 0&\quad 0&\quad 0.08&\quad 0.94&\quad 0&\quad 0&\quad 0\\ 0&\quad 0&\quad 0&\quad 0&\quad 0&\quad 0.06&\quad 0.94&\quad 0&\quad 0\\ 0&\quad 0&\quad 0&\quad 0&\quad 0&\quad 0&\quad 0.06&\quad 0.94&\quad 0\\ 0&\quad 0&\quad 0&\quad 0&\quad 0&\quad 0&\quad 0&\quad 0.06&\quad 0.95 \end{bmatrix}. \end{aligned}$$

The nine stages denote seedlings, saplings I and II, juveniles I–V, and adult trees and the time-steps correspond to years. We refer the reader to Olmsted and Alvarez-Buylla (1995) for details on the phenology of *C. readii*.

The spectral radius of *A* in (5.1) is $1.0549 >1$, so that the uncontrolled population (2.1) is predicted to grow asymptotically. As with the pronghorn example in Sect. 2, we assume that we do not know the entire population distribution at each time-step exactly and again only have access to some part of the state. For simplicity, we consider the case where just two per time-step measurements are made and correspondingly have access to two stages for replenishment. We assume that the seedlings and adult tree stage classes may be restocked and harvested, respectively, leading to the *B* matrix:

$$\begin{aligned} B = \begin{bmatrix}1&\quad 0&\quad 0&\quad 0&\quad 0&\quad 0&\quad 0&\quad 0&\quad 0&\quad 0 \\ 0&\quad 0&\quad 0&\quad 0&\quad 0&\quad 0&\quad 0&\quad 0&\quad 0&\quad 1 \end{bmatrix}^T. \end{aligned}$$

Furthermore, we assume that we are able to measure the abundances of the final two stages; the largest juvenile trees and adult trees so that:

$$\begin{aligned} C = \begin{bmatrix}0&\quad 0&\quad 0&\quad 0&\quad 0&\quad 0&\quad 0&\quad 0&\quad 1&\quad 0 \\ 0&\quad 0&\quad 0&\quad 0&\quad 0&\quad 0&\quad 0&\quad 0&\quad 0&\quad 1 \end{bmatrix}. \end{aligned}$$

The set-point regulation objective is to determine a feedback $F_2$ in (PI2aw) and reference *r* such that the low-gain PI control system (PI2aw) drives the population to some non-zero level, and to determine the resulting adult tree harvest. The input *u*(*t*) is given by:

5.2 $$\begin{aligned} u(t) = \begin{bmatrix}u_1(t) \\ u_2(t) \end{bmatrix} = -F_2Cx(t) + \mathrm{sat}\,(x_c(t)), \quad t \in \mathbb {N}_0, \end{aligned}$$

where $u_1(t)$ denotes the number of seedlings planted at time-step $t \in \mathbb {N}_0$, and is desired to be nonnegative. Similarly, $u_2(t)$ denotes the number of adult trees harvested at time-step $t \in \mathbb {N}_0$, and should be negative. Indeed, we do not want to harvest seedlings or plant adult trees. Roughly speaking, the negative term $-F_2Cx$ on the right hand side of (5.2) determines the harvesting yield and the positive term $\mathrm{sat}\,(x_c)$ from the integral control law determines the replanting scheme.

We require $F_2 \in \mathbb {R}^{2 \times 2}$ such that

5.3 $$\begin{aligned} A_0 := A- B F_2 C \in \mathbb {R}^{9 \times 9}_+ \quad \text {and} \quad r(A_0) <1. \end{aligned}$$

Then, for each $$r = \begin{bmatrix}r_1&r_2 \end{bmatrix}^T = G_{C A_0 B}(1) v \in \mathbb {R}^2_+$$ for $v \in \mathbb {R}^2_+$, the following asymptotic yields are obtained

5.4 $$\begin{aligned}&\text {population distribution:}&x^\infty&= (I-A_0)^{-1} B v, \end{aligned}$$

5.5 $$\begin{aligned}&\text {planting/harvesting effort:}&u^\infty&= - F_2 r + v = (-F_2 G_{CA_0B}(1) + I)v, \end{aligned}$$

5.6 $$\begin{aligned}&\text {measured abundances:}&x_8^*&= r_1 \quad \text {and} \quad x_9^*= r_2 . \end{aligned}$$

First, we construct $F_2 \in \mathbb {R}^{2 \times 2}$ to satisfy (5.3). By considering the product $B F_2 C$, we seek to replace the ninth row of *A* by zero, which necessitates

5.7 $$\begin{aligned} F_2 = \begin{bmatrix}0&\quad 0 \\f_1&\quad f_2 \end{bmatrix} := \begin{bmatrix}0&\quad 0\\ 0.06&\quad 0.95 \end{bmatrix}, \end{aligned}$$

and yields

5.8 $$\begin{aligned}&A_0 = A - B F_2 C\nonumber \\&\quad = \begin{bmatrix}0&\quad 0&\quad 0&\quad 0&\quad 0&\quad 0&\quad 0&\quad 0&\quad 55.8\\ 0.18&\quad 0.8&\quad 0&\quad 0&\quad 0&\quad 0&\quad 0&\quad 0&\quad 0\\ 0&\quad 0.1&\quad 0.89&\quad 0&\quad 0&\quad 0&\quad 0&\quad 0&\quad 0\\ 0&\quad 0&\quad 0.07&\quad 0.94&\quad 0&\quad 0&\quad 0&\quad 0&\quad 0\\ 0&\quad 0&\quad 0&\quad 0.06&\quad 0.92&\quad 0&\quad 0&\quad 0&\quad 0\\ 0&\quad 0&\quad 0&\quad 0&\quad 0.08&\quad 0.94&\quad 0&\quad 0&\quad 0\\ 0&\quad 0&\quad 0&\quad 0&\quad 0&\quad 0.06&\quad 0.94&\quad 0&\quad 0\\ 0&\quad 0&\quad 0&\quad 0&\quad 0&\quad 0&\quad 0.06&\quad 0.94&\quad 0\\ 0&\quad 0&\quad 0&\quad 0&\quad 0&\quad 0&\quad 0&\quad 0&\quad 0 \end{bmatrix}, \quad \text {with } r(A_0) \nonumber \\&\quad = 0.94 <1. \end{aligned}$$

Choosing $F_2$ as in (5.7) satisfies (5.3) from which we compute

$$\begin{aligned} G_{C A_0 B}(1) = \begin{bmatrix}\gamma _1&\quad \gamma _2 \\ 0&\quad 1 \end{bmatrix} := \begin{bmatrix}1.4685&\quad 81.9441 \\ 0&\quad 1 \end{bmatrix}. \end{aligned}$$

It remains to determine *r*, or equivalently *v*. In terms of components the reference $r = G(1)v$ is given by

5.9 $$\begin{aligned} r_1 = \gamma _1 v_1 + \gamma _2 v_2 \quad \text {and} \quad r_2 = v_2. \end{aligned}$$

Of the four quantities $r_1, r_2$, $v_1$ and $v_2$, two are free to be chosen, provided that $0 \le v_1 \le U_1$ and $0 \le v_2 \le U_2$, and the remaining two are determined by (5.9). Rewriting (5.5) in components gives

5.10 $$\begin{aligned} \begin{bmatrix}u_1^\infty \\ u_2^\infty \end{bmatrix}&= \left( -\begin{bmatrix}0&\quad 0 \\f_1&\quad f_2 \end{bmatrix}\begin{bmatrix}\gamma _1&\quad \gamma _2 \\ 0&\quad 1 \end{bmatrix} + \begin{bmatrix}1&\quad 0 \\0&\quad 1 \end{bmatrix}\right) \begin{bmatrix}v_1 \\ v_2 \end{bmatrix} \nonumber \\&\Rightarrow u_1^\infty = v_1 \quad \text {and} \quad u_2^\infty = - f_1 \gamma _1 v_1 + (1- f_1\gamma _2 - f_2)v_2. \end{aligned}$$

Therefore, from (5.10) we see that $v_1 \ge 0$ is the asymptotic replanting level, and from (5.9) that $v_2 = r_2$ is the desired asymptotic adult tree abundance. For given $v_1, v_2$, the expression $u_2^\infty \le 0$ in (5.10) determines the asymptotic number of adult trees harvested per time-step. The asymptotic abundance of the penultimate stage-class is $r_1$. These relations are summarised in Table 2.

**Table 2:** Summary of the roles of $r_1,r_2,v_1$ and $v_2$ in the *C. readii* planting/harvesting example

Quantity	Interpretation
$v_1$	Asymptotic planting level
$v_2 = r_2$	Asymptotic adult tree abundance
$r_1$	Asymptotic final juvenile stage class abundance

**Fig. 4:**
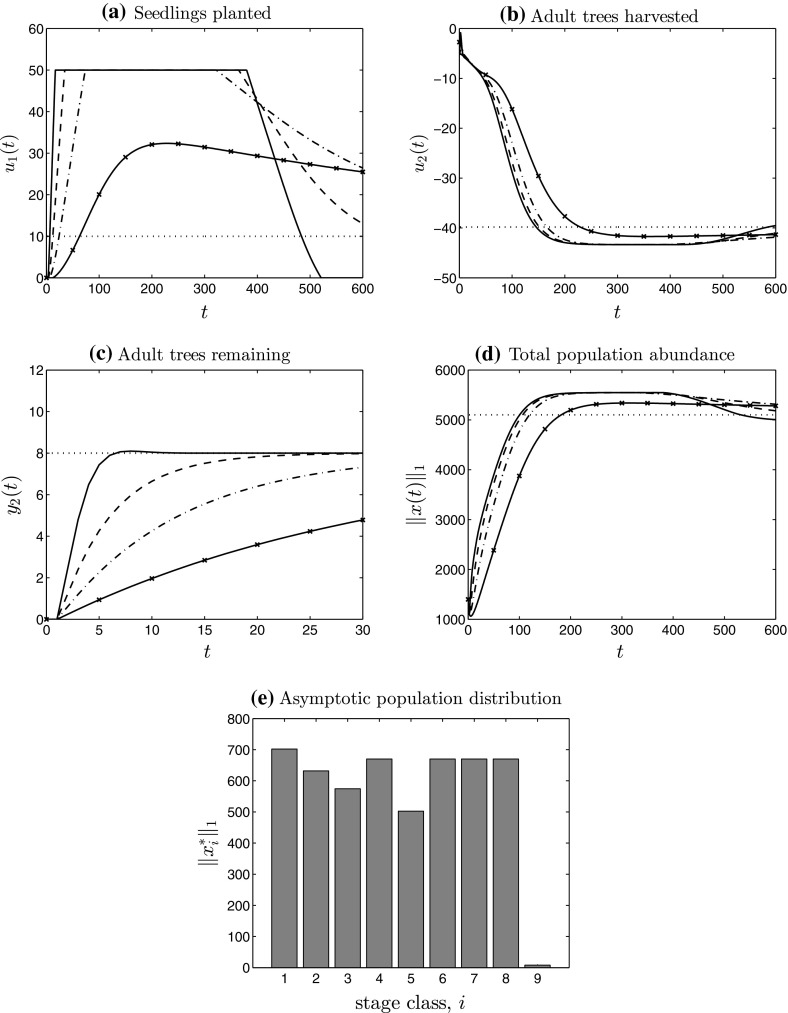
Projections of the replanting and harvesting scheme for the matrix PPM of *C. readii* from Sect. 5. See the main text for more description. In each *plot* the *solid*, *dashed*, *dashed–dotted* and *solid-crossed* correspond to *g* values of 0.01, 0.005, 0.0025 and 0.001, respectively. The *dotted lines* are the references

For the following numerical simulation we suppose an initial population distribution with no adult trees, so that

$$\begin{aligned} x^0 = 2[160\, 120 \, 80 \, 91\, 79 \, 68 \, 57 \, 45 \,0]^T , \end{aligned}$$

As an example, we take

$$\begin{aligned} v = [10 \, 8]^T \Rightarrow r = G(1) v = [670.24 \, 8]^T \quad \text {and} \quad u^\infty = [ 10 \, -39.8]^T, \end{aligned}$$

meaning that, asymptotically, per time-step 10 seedlings are planted, almost 40 adult trees are harvested with eight adult trees remaining in the population. Of course, in practice only an integer number of trees can be harvested per year and we believe that the $39.8 = -u^\infty _2$ is an artefact of the model considered. The asymptotic harvest yield can be altered by tuning $v_1$ and $v_2$ as explained above. To see the role of input saturation, we take

$$\begin{aligned} U = [50 \, 40]^T, \end{aligned}$$

meaning particularly that at most 50 seedlings may be planted per time-step. We repeat the projections for different *g* values, $g \in \{0.01, 0.005,0.0025, 0.001 \}$ and always take

$$\begin{aligned} x_c^0 = 0 \quad \text {and} \quad K = \begin{bmatrix}1&\quad 0 \\ 0&\quad 30 \end{bmatrix}(G(1))^{-1}. \end{aligned}$$

The results are plotted in Fig. 4. The set-point regulation objectives are achieved and the harvest of adult trees increases from zero to (almost) 40 per year, peaking at approximately 43 trees per year. Furthermore, although not specified as a management objective, the total tree abundance rises from $\Vert x^0\Vert _1 = 1400$–5100. We note that the resulting dynamics are rather slow; the time-steps here denote years. This is, in part we suspect, because of the admittedly somewhat limited control actions of only adding to the first stage class and removing from the last. The uncontrolled dynamics themselves are slow as mathematically the matrix *A* has nearly ones on the diagonal and very small entries on the sub diagonal. Biologically, the species *C. readii* is long lived; Olmsted and Alvarez-Buylla (1995) estimate the maximal life span as over 145 years, yet the model is a size based model. That said, the speed of convergence could be increased by allowing more control actions and measurements and adding a ‘larger’ proportional part *F* to the control law. In this case the explanation of the roles of *r* and *v* related by $r = G(1)v$ become more complicated. It is also the case that we have not explored the roles of tuning *K* and *g* further, or of the initial controller state $x_c^0$; all of which can affect the transient dynamics of the model.

To demonstrate robustness of the PI controller, we now assume that the recruitment of the population is not fixed at 55.8, but unknown and denoted by *f*. We have relegated proofs of the subsequent claims to “Appendix C”. If $f \ge 0$ is constant, then owing to the particular structure of this model and the uncertainty, the reference *r* is still tracked asymptotically. This is an example of convergent disturbance rejection, Corollary 4.10. Moreover, a calculation shows that

**Fig. 5:**
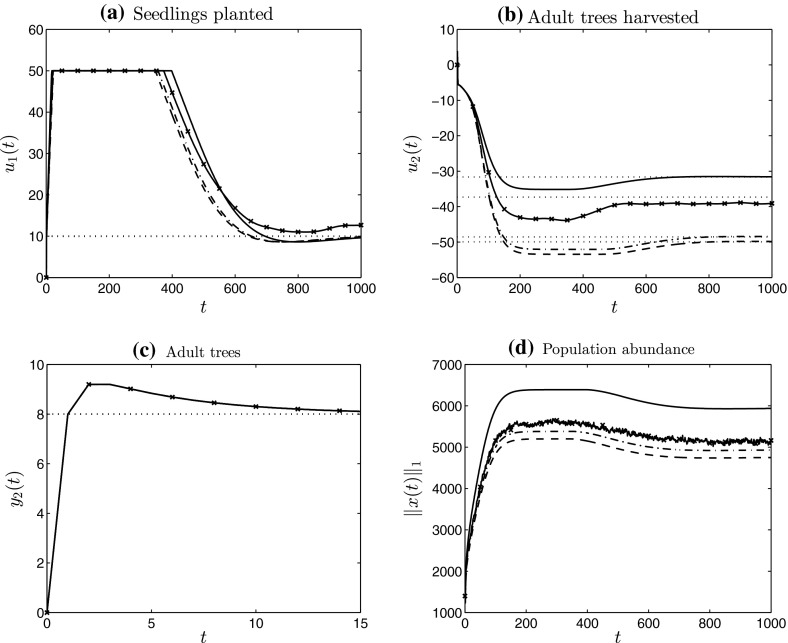
Projections of the replanting and harvesting scheme for the matrix PPM of *C. readii* from Sect. 5 in the presence of model uncertainty. See the main text for more description. In each *plot* the *solid*, *dashed*, *dashed–dotted* correspond to three different and assumed unknown values of $f >0$. The *solid-crossed* correspond to *f*(*t*) drawn from a truncated normal distribution. Here $g=0.05$ is fixed across the simulations. The *dotted lines* are the references

5.11 $$\begin{aligned} G(1) = \begin{bmatrix}\gamma _1&\quad \gamma _1 f \\ 0&\quad 1 \end{bmatrix}, \end{aligned}$$

and hence the relations (5.10) and (5.9) hold with $\gamma _2$ replaced by $\gamma _1 f$. The key interpretations that $v_1$ is the asymptotic planting level and $r_2 = v_2$ is the asymptotic abundance of adult trees hold as before and are thus independent of *f*. Figure 5 contains three simulations with randomly chosen, but positive *f*. Here we have fixed $v_1$ and $v_2$ as before, so that now $r_1$ and the asymptotic harvest yield varies as *f* and thus *G*(1) does.

A more appropriate model may be to consider the situation where *f* is time varying with values $f(t), t \in \mathbb {N}_0$, the inclusion of which reflects environmental or demographic stochasticity. It can be demonstrated that the second output $y_2$, denoting adult trees, still converges to $r_2$. The population abundances *x*, planting/harvesting quantities *u* and abundance of largest juvenile trees need not converge in general. However, the ISS estimate of Proposition 4.9 applies. Figure 5 contains a simulation where *f*(*t*) is drawn from a pseudo-random truncated normal with mean 55.8 and variance 4. We note that, as predicted, the second output, number of adult trees present, rejects the disturbances to the model and is the same across all simulations. $\square$

### *Example 5.2*

**Fig. 6:**
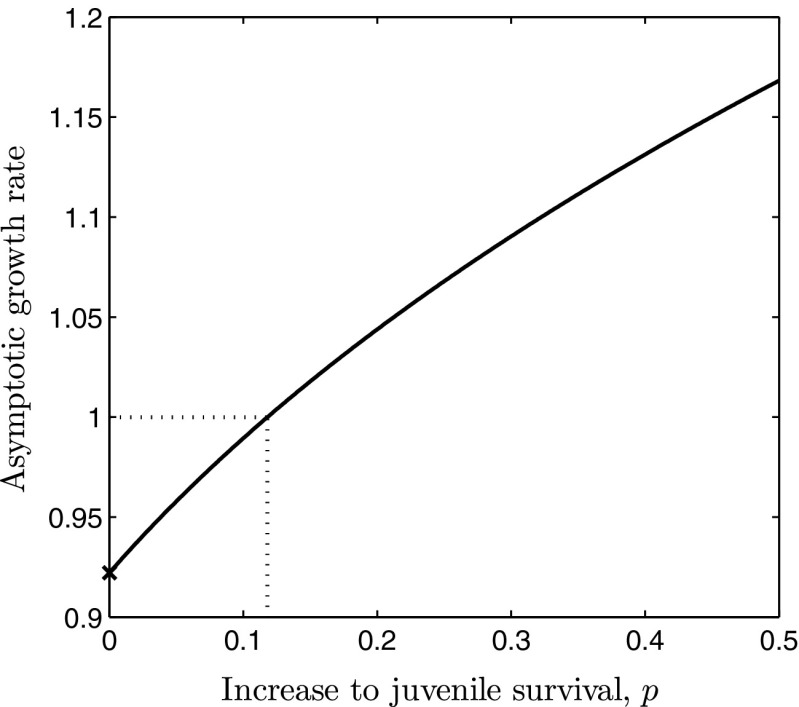
Plot of perturbation to neonate survival *p* against asymptotic growth rate (spectral radius) for the projection matrix model for pronghorn in Example 5.2. The *cross* denotes the unperturbed model and the *dotted line* denotes the perturbation required to achieve a growth rate of one, corresponding to asymptotic persistence

We revisit the matrix projection model for pronghorn from Sect. 2 to demonstrate how low-gain PI control may be combined with other management strategies. The restocking strategy dictated by the low-gain PI controller (Iaw) solves the stated management problem, demonstrated in Fig. 2. However, the asymptotic restocking levels are c. 200 and c. 150 female and male neonates per year, respectively, to maintain a stable population with 120 prime females and 100 prime males. These restocking levels may be too large to implement practically. We suspect that they are so high because the modelled rate of neonate survival and transition to the (next) juvenile stage class is very low, 0.059 in fact (below 6 %). Recall that the uncontrolled population specified by (2.1) has asymptotic rate of decline $0.9222<1$. We investigate the effect of improved neonate survival (of both sexes) *p* on the asymptotic growth rate of the controlled population. Appealing to the perturbation analysis of Hodgson et al. (2006, Theorem 3.3) the relationship in Fig. 6 is obtained between perturbation to survival and resulting asymptotic growth rate. The details are contained in “Appendix A”.

**Fig. 7:**
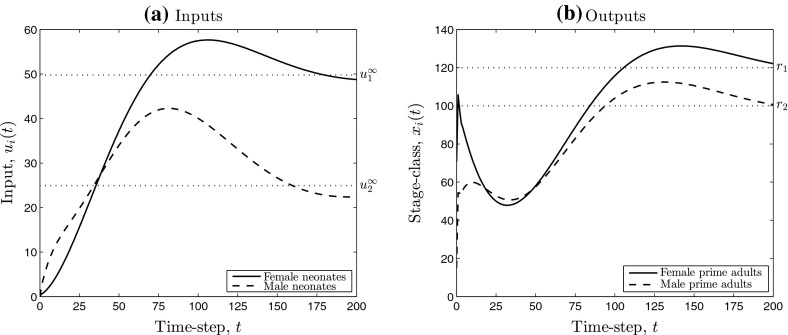
Simulations of the low-gain integral control model (Iaw) applied to the perturbed pronghorn matrix model of Example 5.2. **a**, **b** Contain the inputs and outputs, respectively. The *dotted lines* denote the components of the limiting input (**a**) and the chosen set-point (**b**)

Although a perturbation of only 0.1180 is required to reach population stasis, note that this corresponds to an approximately 200 % increase of current neonate survival, which may also be infeasible to implement. Therefore, to reach the same management objective described in Sect. 2, we explore the combination of the low-gain PI control model (Iaw) with a perturbation of 0.0590 (which is still 100 % of current survival) to neonate survival. Practically, the latter management strategy corresponds to some environmental change. Note that the perturbation to survival alone leads to an asymptotic growth rate of $0.9638 <1$, so is not enough by itself to reverse the predicted asymptotic decline. Simulations of the combined management strategy are plotted in Fig. 7. The demonstrable difference between Figs. 2 and 7 is that in the later the asymptotic restocking rates have fallen to c. 50 and c. 25 female and male individuals per year, respectively. Finally, we note that writing the perturbation to the pronghorn projection matrix *A* as $A + D_1 p D_2$ (with $D_1$ and $D_2$ given by (6.6)), it is possible to see how the predicted asymptotic level of restocking changes with perturbation *p* (provided that $r(A + D_1 p D_2) <1$. Indeed, according to Theorem 4.6 (a), $u^\infty$ is given by

$$\begin{aligned} u^\infty = (C(I-(A + D_1 p D_2))^{-1}B)^{-1}r. \end{aligned}$$

$$\square $$

## Discussion

Low-gain PI control with input saturation has been reconsidered and extended to discrete-time, positive state linear systems where multiple outputs are regulated to desired, necessarily nonnegative, set-points. Our results hold for both finite-dimensional and a class of infinite-dimensional systems. The motivation for the current study is twofold: first, to further explore the utility of feedback control in ecological management type problems, and it is in this context that we have posed much of the present material and our examples. The second purpose is to further develop the suite of robust feedback control for positive state systems—models that arise in a variety of other physically and biologically motivated scenarios.

The present contribution is a sequel to Guiver et al. (2015), where we first considered low-gain PI control as a potential tool for ecological management. There only a single (scalar) per time-step measurement or output of the to-be-controlled system was made, with a view to regulating to a single (scalar) set-point, and thus only a single per time-step control action is required. In other words, Guiver et al. (2015) considered robust regulation of a single management goal. Although conceptually very similar, additional mathematical difficulties arise in extending these results to the natural situation where several management objectives are specified (that is, multi-input, multi-output systems) and additionally in the presence of input saturation to reflect per time-step resource constraints. Specifically, two issues had to be overcome. First, in Sect. 4.1 we described the set of feasible set-points: candidate asymptotic outputs of a positive state linear system. Feasible set-points are subsequently used in our main results, Theorem 4.6 and Corollary 4.7, as the asymptotic limits of the output of a low-gain PI control system. To summarise, Lemma 4.2 states that the set of trackable outputs with positive state includes the nonnegative linear span of the columns of *G*(1), the transfer function evaluated at one. The set of trackable outputs with positive state is enlarged by incorporating a proportional component to the feedback law, Lemma 4.4. Second, in Sect. 4.2 we addressed the problem of including input saturation and still achieving robust set-point regulation. We achieved this by appending a simple anti-windup mechanism (the term involving *E* in (Iaw)) in the integral controller and thereby preventing the destabilising phenomenon associated with input saturation in control theory known as “integrator windup”, discussed in Sect. 4.3. Our main results are Theorem 4.6 and Corollary 4.7 which are low-gain PI control results for positive state systems and mirror the existing, well-known case recorded in Theorem 3.1.

The low-gain PI control system (Iaw) contains demonstrable robustness to certain sources of model uncertainty and disturbances, as described in Sect. 4.4. These facets are a hugely important aspect of feedback control, and a reason why population managers may wish to consider its utility in applications, as ecological models are often highly uncertain. To ensure, however, that (Iaw) is efficacious a sufficiently accurate estimate of *G*(1) is required. A possible fruitful future avenue of research would be to investigate techniques for computing the matrix parameter *E* (which, recall, depends on *G*(1)) adaptively, so that *E* is the output of some dynamic or iterative process. Adaptive control techniques are already known to compute the low-gain parameter $g >0$ adaptively; either in the scalar output case (Logemann and Ryan 2000), or in the multi-input, multi-output case but *without* input saturation (Ke et al. 2009). An adaptive scheme here would ideally determine a suitable *E* without requiring knowledge of *G*(1).

We comment that transfer functions are ubiquitous objects in control theory as they provide a so-called “frequency domain” description of (usually-controlled) dynamic processes. Historically, the term frequency in an engineering context refers to the frequency of oscillation, such as of an electrical alternating current. Intuitively, and amongst other beneficial properties, the frequency domain provides an elegant description of the behaviour of dynamical systems driven by periodic signals and how dynamical systems alter or modulate the phase and amplitude of an incoming periodic signal. Given that numerous physical and biological drivers are (at least roughly) periodic (such as daylight, rainfall or temperature), it is no surprise that a frequency domain approach to ecological modelling has recently been brought to an ecological audience (Greeman and Benton 2005; Worden et al. 2010). Transfer functions have also been employed in ecological modelling in the context of perturbation analysis in Hodgson and Townley (2004), Hodgson et al. (2006) and Stott et al. (2012), as we exploited in Example 5.2 for a modelled pronghorn population. Here the transfer function provides an analytic relationship between perturbations to a population’s life histories and the resulting change to asymptotic growth rate and, in that sense, is a form of sensitivity analysis. We believe that the mature and well-studied language of systems and control theory has much to offer ecological modelling and management. Conversely, the continued study of ecology or ecosystems from a control theory perspective, particularly processes that exhibit feedback structures or feedback-type behaviour may, in the spirit of biomimicry, lead to novel concepts in control theory with other applications.

In closing, we reiterate the distinction between robust control and optimal control. Recall that in the former a control or input is designed to achieve some desired dynamical behaviour *in spite* of uncertainty or disturbances to the dynamics whilst in the latter, a control or input is chosen to achieve some desired dynamic behaviour *while also* minimising a prescribed functional. Broadly speaking (as there are always exceptions), robust control is not optimal and optimal control is not robust. We have explored the use of feedback control for robust ecological management and have not addressed the subject of costs here. As we sought to emphasise in Guiver et al. (2015), inputs obtained from many classical optimal control results are not always robust to various forms of uncertainty. Needless to say, as we believe that ecological models are naturally prone to uncertainty, and indeed as the biological and ecological literature contains numerous papers contributing to the theory and application of optimal control, we have instead focussed on further developing the set of robust feedback control tools for ecological management. We acknowledge the demands placed on population managers by limited resources, and the consequent desire to use those resources wisely. Certainly, more research is required in combining optimal control with robust control in the field of ecological management.

## Appendix A: Model parameters used in examples

*Pronghorn matrix PPM* The matrix model for female pronghorn is based on Berger and Conner (2008, Table 4, wolf-free site) and is

$$\begin{aligned} \begin{bmatrix}0&\quad 0&\quad 0.829 \\ 0.059&\quad 0&\quad 0 \\0&\quad 0.872&\quad 0.872 \end{bmatrix}, \end{aligned}$$

where stage classes one to three denote female neonates, yearlings and prime adults, respectively. We have removed the fourth stage class denoting senescent adults as this stage does not contribute to the life-cycle, and its inclusion results in a reducible matrix.

To include males into the model, we assume that they have the same vital rates as the females, and that the sex-ratio is equal, which leads to the projection matrix

6.1 $$\begin{aligned} A = \begin{bmatrix}0&\quad 0&\quad 0.8290&\quad 0&\quad 0&\quad 0\\ 0.059&\quad 0&\quad 0&\quad 0&\quad 0&\quad 0\\ 0&\quad 0.8720&\quad 0.8720&\quad 0&\quad 0&\quad 0\\ 0&\quad 0&\quad 0.8290&\quad 0&\quad 0&\quad 0\\ 0&\quad 0&\quad 0&\quad 0.059&\quad 0&\quad 0\\ 0&\quad 0&\quad 0&\quad 0&\quad 0.8720&\quad 0.8720 \end{bmatrix}, \end{aligned}$$

in (2.1) and (2.2), where stages one to six now denote female neonates, yearlings, prime adults and male neonates, yearlings, prime adults. The matrix *A* has $r(A) = 0.9222 <1$, thus (A1) holds and the uncontrolled population is predicted to decline asymptotically. The modelling assumption that we are able to independently replenish both neonate stage classes and that we observe both the adult stage classes leads to

6.2 $$\begin{aligned} B = \begin{bmatrix}1&\quad 0\\ 0&\quad 0 \\ 0&\quad 0 \\ 0&\quad 1 \\ 0&\quad 0 \\0&\quad 0 \end{bmatrix} \quad \text {and} \quad C = \begin{bmatrix} 0&\quad 0&\quad 1&\quad 0&\quad 0&\quad 0 \\ 0&\quad 0&\quad 0&\quad 0&\quad 0&\quad 1 \end{bmatrix}. \end{aligned}$$

To verify that the choice of set-point $r = [120 \, 100]^T$ in (2.3) is trackable with positive state, the matrices (*A*, *B*, *C*) as in (6.1) and (6.2), respectively, give rise to

$$\begin{aligned} G(1) = C(I-A)^{-1}B = \begin{bmatrix}0.6028&\quad 0\\ 0.2009&\quad 0.4019 \end{bmatrix}, \end{aligned}$$

and so the asymptotic input

6.3 $$\begin{aligned} u^\infty = (G(1))^{-1}r = \begin{bmatrix}199.0739 \\ 149.3149 \end{bmatrix} , \end{aligned}$$

is required. Since $u^\infty \ge 0$ then evidently $r = G(1)u^\infty \in \langle G(1) \rangle _+$ and hence *r* is trackable with positive state by Lemma 4.2. Obviously, non-integer numbers of individual pronghorns do not make sense and the numbers in (6.3) are an artefact of the non-integers appearing in *A* and would, in practice, of course be rounded to the nearest integer.

To apply a low-gain integral control model (I) to the pronghorn PPM additionally requires that a matrix *K*, a small positive parameter *g* and an initial input $u(0) = x_c^0$ are specified. For the simulations in Fig. 1 we chose:

6.4 $$\begin{aligned} u(0) = \begin{bmatrix}0\\0 \end{bmatrix}, \quad g= 0.01 \quad \text {and} \quad K = \begin{bmatrix} 1.6589&\quad 2.9032\\ -5.1829&\quad 1.0372 \end{bmatrix}. \end{aligned}$$

Recall that *K* and *G*(1) are required to have the property that every eigenvalue of the product *KG*(1) has positive real part [assumption (A2)]. In fact we have chosen *K* deliberately so that

$$\begin{aligned} G(1)K = \begin{bmatrix}1&\quad 1.75 \\ -1.75&\quad 1 \end{bmatrix} =: Q \Rightarrow \sigma (Q) = \{1 \pm 1.75\mathrm {i}\} \subseteq \mathbb {C}_0^+. \end{aligned}$$

Figure 2 contains simulations of the low-gain PI control model with input saturation (Iaw) applied to the pronghorn PPM. To do so per time-step constraints $U_i$ from (4.3) on each input component are required. To ensure that (4.5) holds, each $U_i$ must be no smaller than the respective components of $u^\infty$ in (6.3) and hence we (somewhat arbitrarily) take

6.5 $$\begin{aligned} U := \begin{bmatrix}210 \\ 160 \end{bmatrix}. \end{aligned}$$

For the simulations in Fig. 2 we chose:

$$\begin{aligned} u(0) = \begin{bmatrix}0\\0 \end{bmatrix}, \quad g= 0.004 \quad \text {and} \quad K = \begin{bmatrix} 33.1790&\quad 2.4884\\ -16.58&\quad 48.5155 \end{bmatrix}. \end{aligned}$$

When revisiting the pronghorn model in Example 5.2 we write a perturbation *p* to neonate survival as

6.6 $$\begin{aligned}&\begin{bmatrix}0&\quad 0&\quad 0.8290&\quad 0&\quad 0&\quad 0\\ 0.059 +p&\quad 0&\quad 0&\quad 0&\quad 0&\quad 0\\ 0&\quad 0.8720&\quad 0.8720&\quad 0&\quad 0&\quad 0\\ 0&\quad 0&\quad 0.8290&\quad 0&\quad 0&\quad 0\\ 0&\quad 0&\quad 0&\quad 0.059 +p&\quad 0&\quad 0\\ 0&\quad 0&\quad 0&\quad 0&\quad 0.8720&\quad 0.8720 \end{bmatrix}\nonumber \\&\quad = A + p \begin{bmatrix}0&\quad 0&\quad 0&\quad 0&\quad 0&\quad 0\\ 1&\quad 0&\quad 0&\quad 0&\quad 0&\quad 0\\ 0&\quad 0&\quad 0&\quad 0&\quad 0&\quad 0\\ 0&\quad 0&\quad 0&\quad 0&\quad 0&\quad 0\\ 0&\quad 0&\quad 0&\quad 1&\quad 0&\quad 0\\ 0&\quad 0&\quad 0&\quad 0&\quad 0&\quad 0 \end{bmatrix} = A + p \begin{bmatrix}0&\quad 0\\ 1&\quad 0\\ 0&\quad 0\\ 0&\quad 0\\ 0&\quad 1\\ 0&\quad 0 \end{bmatrix} \begin{bmatrix}1&\quad 0&\quad 0&\quad 0&\quad 0&\quad 0\\ 0&\quad 0&\quad 0&\quad 1&\quad 0&\quad 0 \end{bmatrix}\nonumber \\&\quad =: A + pD_1 D_2. \end{aligned}$$

Hodgson et al. (2006, Theorem 3.3) states that for $\lambda \not \in \sigma (A)$, $\lambda \in \sigma (A+pD_1D_2)$ if, and only if, $1 \in \sigma (p D_2(\lambda I -A)^{-1}D_1)$. When unravelled the latter condition is equivalent to

6.7 $$\begin{aligned} 1 = \frac{90361\times 10^3p}{1.25\times 10^8\lambda ^3 - 1.09\times 10^8\lambda ^2 - 5331299}. \end{aligned}$$

For *p* in the interval [0, 0.5), Eq. (6.7) is solved for $\lambda$ (seeking the largest positive solution denoting $r(A+pD_1D_2)$) and plotted in Fig. 6. The simulations of (Iaw) applied to the pronghorn PPM with *A* replaced by $A + 0.059D_1D_2$ in Fig. 7 were conducted with parameters:

$$\begin{aligned} u(0) = \begin{bmatrix}0\\0 \end{bmatrix}, \quad g= 0.003 \quad \text {and} \quad K = \begin{bmatrix} 0.415&\quad 0 \\ -0.5802&\quad 1.244 \end{bmatrix}. \end{aligned}$$

$$\square $$

*Example* 5.1*continued* For the robustness arguments we first replace the $(1,9){\mathrm{th}}$ entry of *A* in (5.1) by $f >0$ and let ${\hat{f}} = 55.8$ so that the original *A* is denoted by ${\hat{A}}$. Therefore

$$\begin{aligned} A = {\hat{A}} + e_1 (f-{\hat{f}}) e_9^T =: {\hat{A}} + e_1 \delta e_9^T , \end{aligned}$$

where $e_i$ is the $i{\mathrm{th}}$ standard basis vector, and thus also

$$\begin{aligned} A_0 := A - BFC = {\hat{A}} -BFC + e_1 \delta e_9^T =: {\hat{A}}_0 + e_1 \delta e_9^T . \end{aligned}$$

Appealing to the block structure of $A_0$, it follows that $\sigma (A_0) = \sigma ({\hat{A}}_0)$ is independent of *f*. Consequently, *A* satisfies (A) for every $f >0$. Similarly, from (5.11) a calculation shows that

$$\begin{aligned} G(1) = C(I -A)^{-1}B = \begin{bmatrix} \gamma _1&\quad \gamma _1 f \\ 0&\quad 1 \end{bmatrix}, \end{aligned}$$

and hence the known choice

$$\begin{aligned} K = \begin{bmatrix}1&\quad 0 \\ 0&\quad 30 \end{bmatrix}{\hat{G}}(1)^{-1} = \begin{bmatrix}\gamma _1^{-1}&\quad - \gamma _1 {\hat{f}} \\ 0&\quad 30 \end{bmatrix}, \end{aligned}$$

is such that *K* and *G*(1) satisfy (A2) for every $f >0$. We claim that the $y_2$ dynamics, which recall are those of the $9{\mathrm{th}}$ stage-class, are independent of $\delta$. To see this we note that the dynamics for the state *x* are

$$\begin{aligned} x(t+1) = {\hat{A}}_0 x(t) + B \mathrm{sat}\,(x_c(t)) + e_1 e_9^T \delta x(t), \quad x(0) = x^0, \, t \in \mathbb {N}_0, \end{aligned}$$

which by inspection of the 9${\mathrm{th}}$ component yields that

$$\begin{aligned} y_2(t) = x_9(t) = \mathrm{sat}\,_{U_2}{(x_c^{(2)}(t))}. \end{aligned}$$

Moreover, from the integral control update law

$$\begin{aligned} x_c^{(2)}(t+1)&= x_c^{(2)}(t) + 30g(r_2 - x_9(t)) - 30g(x_c^{(2)}(t) - \mathrm{sat}\,_{U_2}{(x_c^{(2)}(t))}) \\&= \underbrace{(1- 30g)}_{<1}x_c^{(2)}(t) + gr_2, \quad t \in \mathbb {N}_0, \end{aligned}$$

provided $g <1/30$, where $x_c^{(2)}(t)$ denotes the second component of $x_c(t)$. Therefore, $x_c^{(2)}(t)$ and thus $y_2(t)$ converge as $t \rightarrow \infty$ (the latter to $r_2$), independently of $\delta$. The above argument holds if $f= f(t)$ is time-varying. Now writing

$$\begin{aligned} A(t) = {\hat{A}} + e_1 (f(t)-{\hat{f}}) e_9^T = {\hat{A}} + B \begin{bmatrix}0&\quad f(t)- {\hat{f}} \\ 0&\quad 0 \end{bmatrix} C =: {\hat{A}} + B {\Delta }(t) C, \end{aligned}$$

the whole state dynamics are given by

$$\begin{aligned} x(t+1)&= A(t) x(t) + Bu(t) = Ax(t) - BFCx(t) + B \mathrm{sat}\,(x_c(t))\\&= {\hat{A}}_0 x(t) + B \mathrm{sat}\,(x_c(t)) + B {\Delta }(t) Cx(t) \\&= {\hat{A}}_0 x(t) + B \mathrm{sat}\,(x_c(t)) + B \underbrace{ \begin{bmatrix}(f(t)-{\hat{f}}) y_2(t) \\ 0 \end{bmatrix}}_{=:d(t)}, \quad x(0) = x^0, \, t \in \mathbb {N}_0. \end{aligned}$$

If *f*(*t*) is constant then *d*(*t*) is convergent, and hence the disturbance to *x*(*t*) is rejected by the output, by Corollary 4.10. If *f*(*t*) is time-varying then the ISS estimates of Proposition 4.9 apply. $\square$

## Appendix B: The *Z*-transform, transfer functions and convolutions

We collect more notation that shall be required for some of the proofs. First, for $\mathcal {B}$ a Banach space with norm $\Vert \cdot \Vert$ and $p \in [1, \infty ]$ we let $\ell ^p = \ell ^p(\mathbb {N}_0; \mathcal {B})$ denote the usual sequence space of $\mathcal {B}$-valued sequences *v* such that

$$\begin{aligned} \Vert v \Vert _{\ell ^p}= & {} \Vert v \Vert _p := \left( \sum _{j\in \mathbb {N}_0}\Vert v(j) \Vert ^p \right) ^{\tfrac{1}{p}} < \infty , \quad p \in (1, \infty ) \quad \text {or}\\ \Vert v \Vert _{\ell ^\infty }= & {} \Vert v \Vert _\infty = \sup _{t \in \mathbb {N}_0} \Vert v(t) \Vert < \infty , \end{aligned}$$

For each sequence *v*, $t \in \mathbb {N}_0$ and $p\in [1, \infty )$, the quantity $\Vert v \Vert _{\ell ^p(0,t)}$ denotes

$$\begin{aligned} \left( \sum _{j =0}^t\Vert v(j) \Vert ^p \right) ^{\tfrac{1}{p}}, \end{aligned}$$

with an analogous definition for $\Vert v \Vert _{\ell ^\infty (0,t)}$. If $\mathcal {H}$ is a Hilbert space with norm $\Vert \cdot \Vert$ induced from an inner-product, then $\ell ^2(\mathbb {N}_0;\mathcal {H})$ is itself a Hilbert space. For a sequence $v \in \ell ^2$, the Z-transform of *v*, denoted ${\hat{v}}$, is a $\mathcal {H}$-valued function of a complex variable given by

7.1 $$\begin{aligned} z \mapsto {\hat{v}}(z) = \sum _{j \in \mathbb {N}_0}\frac{v(j)}{z^j}, \quad z \in \mathbb {C}, \end{aligned}$$

defined wherever the summation converges absolutely, and can be thought of as a discrete-time Laplace transform. We let $\mathbb {E}: = \{ z \in \mathbb {C}:\vert z \vert >1\}$ denote the exterior of the unit complex disc and circle and let $H^2 = H^2(\mathbb {E}; \mathcal {H})$ denote the Hardy space of bounded, analytic $\mathcal {H}$-valued functions on $\mathbb {E}$ with finite Hardy norm

$$\begin{aligned} \Vert w \Vert _{H^2} = \sup _{r > 1} \left( \int _{\vert z \vert =1} \Vert w(r z) \Vert ^2 \, dz \right) ^{\tfrac{1}{2}}. \end{aligned}$$

If $v \in \ell ^2$ then ${\hat{v}} \in H^2$ and furthermore the Parseval equivalence of norms holds

7.2 $$\begin{aligned} \Vert u \Vert _2 = \Vert {\hat{u}} \Vert _{H_2}, \quad \forall u \in \ell ^2. \end{aligned}$$

The above claims are well-known see; for example, Staffans (2005, p. 699).

If $r(A)<1$ and $u \in \ell ^2$ then applying the Z-transform to (2.2) and eliminating ${\hat{x}}(z)$ yields that

7.3 $$\begin{aligned} {\hat{y}}(z) = C(zI-A)^{-1} x^0 + C(zI-A)^{-1}B {\hat{u}}(z) = C(zI-A)^{-1} x^0 + G(z) {\hat{u}}(z),\nonumber \\ \end{aligned}$$

so that when $x^0 = 0$

7.4 $$\begin{aligned} {\hat{y}}(z) = G(z) {\hat{u}}(z), \quad \forall z \in \mathbb {E}. \end{aligned}$$

For two sequences *u*, *v* we let $u *v$ denote the (discrete) convolution of *u* and *v*, with terms given by

$$\begin{aligned} (u *v)(t) := \sum _{j =0 }^t u(t-j) v(j), \quad t \in \mathbb {N}_0, \end{aligned}$$

and record the following fact regarding the Z-transform of convolutions

7.5 $$\begin{aligned} \widehat{(u *v)}(z) = {\hat{u}}(z) \cdot {\hat{v}}(z), \quad \forall u,v \in \ell ^2, \; \forall z \in \mathbb {E}. \end{aligned}$$

We shall also require the following $\ell ^2$ and pointwise estimates for convolutions, respectively

7.6 $$\begin{aligned} \Vert u *v \Vert _2 \le \Vert u\Vert _1 \cdot \Vert v \Vert _2 , \quad \forall u \in \ell ^1, \; \forall v \in \ell ^2. \end{aligned}$$

and

7.7 $$\begin{aligned} \Vert (u *v)(t) \Vert \le \Vert u \Vert _2 \cdot \Vert v \Vert _2 , \quad \forall u,v \in \ell ^2, \; \forall t \in \mathbb {N}_0. \end{aligned}$$

A proof of (7.6) may be found in Desoer and Vidyasagar (1975, p. 244) and (7.7) follows from the Cauchy–Schwarz inequality (equivalently, the Hölder inequality with exponents $p = q = 2$). Let $\mathcal {X}$ denote a Banach space and

$$\begin{aligned} A :\mathcal {X}\rightarrow \mathcal {X}, \quad B :\mathbb {R}^s \rightarrow \mathcal {X}, \quad C:\mathcal {X}\rightarrow \mathbb {R}^s, \end{aligned}$$

denote bounded, linear operators with $r(A) <1$. Then the function

$$\begin{aligned} z \mapsto G(z) = C(zI - A)^{-1} B, \quad z \in \mathbb {E}, \end{aligned}$$

(as defined in (3.2)) is equal to the Z-transform of the sequence *h* defined by

7.8 $$\begin{aligned} h(t) = CA^tB :\mathbb {R}^m \rightarrow \mathbb {R}^m, \quad t \in \mathbb {N}_0, \end{aligned}$$

that is,

7.9 $$\begin{aligned} {\hat{h}}(z) = G(z), \quad \forall z \in \mathbb {E}. \end{aligned}$$

Since $r(A) <1$ it follows that $h \in \ell ^p(\mathbb {N}_0;\mathbb {R}^{m\times m})$ for every $p \ge 1$. Furthermore, combining (7.2), (7.5) and (7.9) we obtain the crucial estimate for $u \in \ell ^2$ and *h* as in (7.8)

7.10 $$\begin{aligned} \Vert h *u \Vert _2 \!=\! \Vert \widehat{(h *u)} \Vert _{H^2} \!=\! \Vert {\hat{h}} \cdot {\hat{u}} \Vert _{H^2} \!=\! \Vert G \cdot {\hat{u}} \Vert _{H^2} \le \Vert G \Vert _\infty \cdot \Vert {\hat{u}} \Vert _{H^2} \!=\! \Vert G \Vert _\infty \cdot \Vert u \Vert _2.\nonumber \\ \end{aligned}$$

For any finitely nonzero sequence *v*, $v = P_T v$, for some $T \in \mathbb {N}$ where $P_T$ is the truncation operator

Clearly, $P_T v \in \ell ^2$ for every $T \in \mathbb {N}$, and applying estimate (7.10) above yields the truncated version

7.11 $$\begin{aligned} \Vert h *v \Vert _{\ell ^2(0,T)} \le \Vert G \Vert _\infty \cdot \Vert v \Vert _{\ell ^2(0,T)}, \end{aligned}$$

that we shall also require for the proofs in the following “Appendix C”.

## Appendix C: Proofs of results

### *Proof of Lemma 4.2*

- Since by assumption $r(A)<1$, the equality
  $$\begin{aligned} (I-A)^{-1} = \sum _{j \in \mathbb {N}_0} A^j, \end{aligned}$$
  holds (and the Neumann series converges absolutely) and thus as *A*, *B* and *C* are nonnegative
  $$\begin{aligned} G_{CAB}(1) = \sum _{j \in \mathbb {N}_0} CA^j B \in \mathbb {R}^{p \times m}_+, \quad \text {as required.} \end{aligned}$$
- A useful ingredient in the following proof is that with $A = A_1 + BF$, since $B,F \ge 0$ it follows that $A_1 \le A$ and so [by, for example, Berman and Plemmons (1994, p. 27)]
  8.1 $$\begin{aligned} r(A_1) \le r(A) <1. \end{aligned}$$

A consequence of (8.1) is that $G_{C A_1 B}(1)$ is well-defined and from (a), $G_{C A_1 B}(1) \in \mathbb {R}^{p \times m}_+$. Choose $r \in \mathrm{im}_+ G_{CA_1 B}(1)$, so that there exists $u_+ \in \mathbb {R}^m_+$ such that

$$\begin{aligned} r = G_{CA_1 B}(1)u_+. \end{aligned}$$

Consider the state–feedback input

8.2 $$\begin{aligned} u(t) := -Fx(t) + u_+, \quad t \in \mathbb {N}_0, \end{aligned}$$

which when inserted into (2.2) gives rise to the closed–loop system

8.3 $$\begin{aligned} x(t+1) = Ax(t) + B u(t) = A_1 x(t) + B u_+, \quad t \in \mathbb {N}_0. \end{aligned}$$

Note that as $A_1, B, u_+ \ge 0$ it follows from (8.3) that $x(t) \ge 0$ for each $t \in \mathbb {N}_0$. Invoking (8.1) yields that *x* is convergent, with limit

8.4 $$\begin{aligned} x^\infty = (I - A_1)^{-1}B u_+. \end{aligned}$$

Consequently, the input *u* given by (8.2) is also convergent as is the output *y*, which therefore satisfies

$$\begin{aligned} \lim _{t \rightarrow \infty } y(t) = \lim _{t \rightarrow \infty } Cx(t) = Cx^\infty = C(I-A_1)^{-1}Bu_+ = G_{CA_1 B}(1)u_+ = r, \end{aligned}$$

whence $r \in \mathbb {R}^p_+$ is trackable with positive state. $\square$

### *Proof of Proposition 4.3*

It suffices to prove that the set of trackable outputs of (*A*, *B*, *C*) with positive state is contained in $\langle G_{C A_1B}(1) \rangle _+$, as the converse inclusion was established in Lemma 4.2 (b). Assume that $r \in \mathbb {R}^p_+$ is trackable with positive state, so that there exists a convergent input *u* (not necessarily nonnegative) such that the state *x*(*t*) is nonnegative for each $t \in \mathbb {N}_0$ and furthermore the output converges to *r*. Thus for each $t \in \mathbb {N}_0$

8.5 $$\begin{aligned} 0 \le x(t+1) = Ax(t) + Bu(t) = A_1 x(t) + B(u(t) + Fx(t)), \end{aligned}$$

so that by assumption (H), $u(t) + Fx(t) \ge 0$. Furthermore as *u*, and thus *x*, are convergent

$$\begin{aligned} u(t) + Fx(t) \rightarrow {\hat{u}} \ge 0, \quad \text {as }t \rightarrow \infty , \end{aligned}$$

yielding that

$$\begin{aligned} r = \lim _{t \rightarrow \infty } Cx(t) = C(I-A_1)^{-1}B {\hat{u}} \in \langle G_{CA_1 B}(1)\rangle _+. \end{aligned}$$

$$\square $$

### *Proof of Lemma 4.4*

- It is well-known [see, for example, Hodgson et al. (2006, Theorem 3.3)] that
  $$\begin{aligned} 1 \in \sigma ( A_1 +BF) \iff 1 \in \sigma (G_{FA_1 B}(1)) \iff 0 \in \sigma (I - G_{FA_1 B}(1)). \end{aligned}$$
  As $r(A_1 +BF) = r(A) <1$, the above equivalences yield that $0 \not \in \sigma (I - G_{FA_1 B}(1))$, proving the claim.
- A calculation using the Sherman–Woodbury–Morrison Formula [see, for example, Hager (1989)] gives that
  8.6 $$\begin{aligned} G_{CAB}(1)&= C(I-A)^{-1}B = C((I-A_1) - BF)^{-1}B \nonumber \\&= G_{C A_1B}(1) + G_{C A_1B}(1) [ I - G_{F A_1B}(1)]^{-1}G_{F A_1B}(1)\nonumber \\&= G_{C A_1B}(1)[ I - G_{F A_1B}(1)]^{-1} \nonumber \\&= G_{C A_1B}(1) \sum _{k\in \mathbb {N}_0} (G_{F A_1B}(1))^k, \end{aligned}$$
  where we have used part (a) for the existence of the inverse of $I - G_{F A_1B}(1)$. Claim (b) follows from (8.6) once we note that the Neumann series appearing in (8.6) is nonnegative.

$$\square $$

### *Proof of Theorem 4.6*

The choice of *r* in (4.5) ensures that there exists $v \in \mathbb {R}^m$ such that

8.7 $$\begin{aligned} r = G(1) \mathrm{sat}\,(v). \end{aligned}$$

We note that *v* need not be unique. From its definition, the saturation function has the idempotent property that

8.8 $$\begin{aligned} \mathrm{sat}\,(\mathrm{sat}\,(w)) = \mathrm{sat}\,(w), \quad \forall w \in \mathbb {R}^m. \end{aligned}$$

We define the shifted function $\widetilde{\mathrm{sat}}\,:\mathbb {R}^m \rightarrow \mathbb {R}^m$ by

8.9 $$\begin{aligned} \widetilde{\mathrm{sat}}\, :\mathbb {R}^m \rightarrow \mathbb {R}^m, \quad \widetilde{\mathrm{sat}}\,(w) := \mathrm{sat}\,(w + \mathrm{sat}\,(v)) - \mathrm{sat}\,(v), \end{aligned}$$

which from (8.8) satisfies $\widetilde{\mathrm{sat}}\,(0) =0$. Introduce the shifted co-ordinates ${\tilde{x}}$ and ${\tilde{x}}_c$ by

8.10a $$\begin{aligned} {\tilde{x}} (t)&:= x(t) - x^\infty , \quad t \in \mathbb {N}_0, \end{aligned}$$

8.10b $$\begin{aligned} \text {and} \quad {\tilde{x}}_c (t)&:= x_c(t) - \mathrm{sat}\,(v), \quad t \in \mathbb {N}_0, \end{aligned}$$

where $x^\infty$ is as in Theorem 4.6 (b). For notational convenience we introduce the (so-called deadzone) nonlinearity ${\varPsi }$ by

8.11 $$\begin{aligned} {\varPsi } :\mathbb {R}^m \rightarrow \mathbb {R}^m, \quad {\varPsi }(w) := w - \widetilde{\mathrm{sat}}\,(w), \end{aligned}$$

which, it is routine to verify, satisfies the linear estimate

8.12 $$\begin{aligned} \Vert {\varPsi }(w) \Vert \le \Vert w \Vert , \quad \forall w \in \mathbb {R}^m. \end{aligned}$$

An elementary sequence of calculations shows that ${\tilde{x}}$ and ${\tilde{x}}_c$ have dynamics given by

8.13 $$\begin{aligned} \begin{bmatrix}{\tilde{x}} (t+1) \\ {\tilde{x}}_c (t+1) \end{bmatrix} = \begin{bmatrix}A&B \\ -gKC&I \end{bmatrix}\begin{bmatrix}{\tilde{x}} (t) \\ {\tilde{x}}_c (t) \end{bmatrix} - \begin{bmatrix}B \\ E \end{bmatrix}{\varPsi }\left( \begin{bmatrix}0&I \end{bmatrix}\begin{bmatrix}{\tilde{x}} (t) \\ {\tilde{x}}_c (t) \end{bmatrix}\right) , \quad t \in \mathbb {N}_0,\nonumber \\ \end{aligned}$$

which, after introducing

8.14 $$\begin{aligned} \xi := \begin{bmatrix}{\tilde{x}} \\ {\tilde{x}}_c \end{bmatrix}, \quad \mathcal {A}:= \begin{bmatrix}A&B \\ -gKC&I \end{bmatrix}, \quad \mathcal {B}:= - \begin{bmatrix}B \\ E \end{bmatrix} \quad \text {and} \quad \mathcal {C}:= \begin{bmatrix}0&I \end{bmatrix}, \end{aligned}$$

is rewritten as

8.15 $$\begin{aligned} \xi (t+1) = \mathcal {A}\xi (t) + \mathcal {B}{\varPsi }(\mathcal {C}\xi (t)), \quad \xi (0) = \xi _0, \quad t \in \mathbb {N}_0. \end{aligned}$$

Our aim is to demonstrate that our choice of $E = gKG(1) \in \mathbb {R}^{p\times m}$ in (4.4) ensures that zero is a globally asymptotically stable equilibrium of (8.15) for all sufficiently small, but positive, *g*. To that end, as in Theorem 3.1 (see Logemann and Townley 1997, Theorem 2.5, Remark 2.7), assumptions (A1) and (A2) (particularly the choice of *K*) imply that there exists ${\hat{g}} >0$ such that

8.16 $$\begin{aligned} r(\mathcal {A}) <1, \quad \forall g \in (0, {\hat{g}}). \end{aligned}$$

For such $g \in (0, {\hat{g}})$, we consider the transfer function $\mathcal {G}_g$ of the triple $(\mathcal {A},\mathcal {B},\mathcal {C})$, which is given by

$$\begin{aligned} z \mapsto \mathcal {G}_g(z) \!=\! \mathcal {C}(zI - \mathcal {A})^{-1} \mathcal {B}\!=\! -\! \begin{bmatrix}0&I \end{bmatrix}\begin{bmatrix}zI - A&\quad -B \\ gKC&\quad (z-1)I \end{bmatrix}^{-1}\begin{bmatrix}B \\ E \end{bmatrix}, \quad \! z \!\in \! \mathbb {C}, \vert z \vert \!\ge \! 1. \end{aligned}$$

By using blockwise inversion and subsituting our choice of $E = gKG(1)$, it follows that $\mathcal {G}_g$ reduces to

8.17 $$\begin{aligned} \mathcal {G}_g(z) = ((z-1)I + gKG(z))^{-1} (gKG(z) - gKG(1)) . \end{aligned}$$

We seek to establish the following claim: there exists a $g^*\in (0, {\hat{g}})$ and $\rho \in (0,1)$ such that for all $g \in (0, g^*)$

8.18 $$\begin{aligned} \Vert \mathcal {G}_g \Vert _\infty \le \rho \, . \end{aligned}$$

In what follows we let $\mathbb {T}$ denote the complex unit circle $\mathbb {T}= \{ z \in \mathbb {C}:\vert z \vert =1\}$. We note that for every $g \in (0, {\hat{g}})$ and $z \in \mathbb {T}$, $zI -A$ is invertible (as $r(A) <1$), as is

$$\begin{aligned} \begin{bmatrix}zI -A&\quad -B \\ gKC&\quad (z-1)I \end{bmatrix}, \end{aligned}$$

and hence by, for example, Zhang (2005, Theorem 1.2), it follows that the Schur complement

$$\begin{aligned} T(z) := (z-1)I + gKG(z), \end{aligned}$$

is invertible as well. For $z \in \mathbb {T}$ we define

$$\begin{aligned} Q(z) := gK(G(z) - G(1)), \end{aligned}$$

so that

8.19 $$\begin{aligned} \mathcal {G}_g(z) = T(z)^{-1} Q(z), \quad z \in \mathbb {T}. \end{aligned}$$

Consider the following chain of equivalences: fix $\rho \in (0,1)$

$$\begin{aligned} \Vert \mathcal {G}_g \Vert _\infty \le \rho&\iff \sup _{z \in \mathbb {T}} \Vert \mathcal {G}_g(z) \Vert \le \rho \iff \Vert \mathcal {G}_g(z) \Vert \le \rho \quad \forall z \in \mathbb {T},\\&\iff \Vert (\mathcal {G}_g(z))^*\Vert \le \rho \quad \forall z \in \mathbb {T}, \end{aligned}$$

where superscript ${}^*$ denotes the Hilbert space adjoint operator, and we have used in the last equivalence that a bounded operator on a Hilbert space has the same operator norm as its adjoint. Therefore

8.20 $$\begin{aligned} \Vert \mathcal {G}_g \Vert _\infty \le \rho&\iff \Vert (\mathcal {G}_g(z))^*u \Vert ^2 \le \rho ^2 \Vert u \Vert ^2, \quad \forall z \in \mathbb {T}, \; \forall u \in \mathbb {C}^m. \end{aligned}$$

Expressing the right hand side of (8.20) in terms of the inner-product $\langle \cdot , \cdot \rangle$ on $\mathbb {C}^m$ and using the decomposition (8.19) yields that

where we have set $v = (T(z))^{-*}u$, and noted that as *T*(*z*) is bijective, $v \in \mathbb {C}^m$ ranges across all of $\mathbb {C}^m$ as $u \in \mathbb {C}^m$ does. Hence,

8.21 $$\begin{aligned} \Vert \mathcal {G}_g \Vert _\infty \le \rho&\iff \Vert (Q(z))^*v\Vert ^2 \le \rho ^2\Vert (T(z))^*v \Vert ^2, \quad \forall z \in \mathbb {T}, \; \forall v \in \mathbb {C}^m, \nonumber \\&\iff \Vert (Q(z))^*v\Vert \le \rho \Vert (T(z))^*v \Vert , \quad \forall z \in \mathbb {T}, \; \forall v \in \mathbb {C}^m. \end{aligned}$$

We seek to establish that the right hand side of (8.21) holds, which, written out in full claims that there exists $g^*>0$ and $\rho \in (0, 1)$ such that for all $g \in (0, g^*)$

8.22 $$\begin{aligned} \Vert g(KG(z) - KG(1))^*v \Vert \le \rho \Vert [({\overline{z}} - 1)I + g(KG(z))^*] v \Vert , \quad \forall z \in \mathbb {T}, \; \forall v \in \mathbb {C}^m,\nonumber \\ \end{aligned}$$

where for $w \in \mathbb {C}$, $\overline{w}$ denotes its complex conjugate. The arguments that follow are based on those used in the proof of Logemann and Townley (1997, Theorem 2.5), although adapted for our purposes. Seeking a contradiction, suppose that the above claim is false. Therefore, there exist sequences $(g_n)_{n \in \mathbb {N}} \subseteq (0, \infty )$, $(z_n)_{n \in \mathbb {N}} \subseteq \mathbb {T}$ and $(v_n)_{n \in \mathbb {N}} \subseteq \mathbb {C}^m$ such that $g_n \searrow 0$ as $n \rightarrow \infty$, but

8.23 $$\begin{aligned} \Vert g_n(KG(z_n) \!-\! KG(1))^*v_n \Vert \!>\! \left( 1-\frac{1}{n}\right) \Vert [(\overline{z_n} \!-\! 1)I \!+\! g_n(KG(z_n))^*] v_n \Vert , \, \forall n \in \mathbb {N}.\nonumber \\ \end{aligned}$$

The inequality (8.23) necessitates that $v_n \ne 0$ for each $n \in \mathbb {N}$ and so (by multiplying both sides of (8.23) by a positive constant if necessary) we may assume that

8.24 $$\begin{aligned} \Vert v_n \Vert =1, \quad n \in \mathbb {N}. \end{aligned}$$

Arguing similarly, inequality (8.23) also necessitates that $z_n \ne 1$ for each $n \in \mathbb {N}$ and, as $z_n \in \mathbb {T}$, it follows that

8.25 $$\begin{aligned} \mathrm{Re}\, z_n <1, \quad \forall n \in \mathbb {N}. \end{aligned}$$

Since the sequences $(z_n)_{n \in \mathbb {N}} \subseteq \mathbb {T}$ and $(v_n)_{n \in \mathbb {N}} \subseteq \mathbb {C}^m$ are both bounded, we may pass to a subsequence (not relabelled) along which both $(z_n)_{n \in \mathbb {N}} \subseteq \mathbb {T}$ and $(v_n)_{n \in \mathbb {N}} \subseteq \mathbb {C}^m$ converge. We denote the limits of these sequences by $z^\infty \in \mathbb {T}$ and $v^\infty \in \mathbb {C}^m$, respectively, and note that $(\overline{z_n})_{n \in \mathbb {N}}$ has limit $\overline{z^\infty }$. The equalities (8.24) imply that

$$\begin{aligned} \Vert v^\infty \Vert = 1 \quad \text {and}\quad \text {so} \quad v^\infty \ne 0. \end{aligned}$$

Since $(KG(z_n))_{n \in \mathbb {N}}$ is bounded, taking the limit $n \rightarrow \infty$ in (8.23) yields that

$$\begin{aligned} 0 \ge \Vert (\overline{z^\infty } -1)v^\infty \Vert = \vert \overline{z^\infty } -1 \vert \cdot \Vert v^\infty \Vert = \vert \overline{z^\infty } -1 \vert \ge 0, \end{aligned}$$

and hence $\overline{z^\infty } =1 = z^\infty$. Dividing both sides of (8.23) by $g_n >0$, we obtain the following estimates for $n \in \mathbb {N}$, $n \ge 2$

8.26 $$\begin{aligned} \Vert (KG(z_n) - KG(1))^*v_n\Vert&> \left( 1-\frac{1}{n}\right) \left\| \left[ \frac{\overline{z_n} - 1}{g_n}I + (KG(z_n))^*\right] v_n \right\| , \nonumber \\&\ge \frac{1}{2}\left\| \left[ \frac{\overline{z_n} - 1}{g_n}I + (KG(z_n))^*\right] v_n \right\| , \nonumber \\&\ge \frac{1}{2}\left\| \left( \frac{\overline{z_n} - 1}{g_n}\right) v_n \right\| - \frac{1}{2}\Vert (KG(z_n))^*v_n \Vert , \end{aligned}$$

so that

8.27 $$\begin{aligned} 0\le & {} \left| \frac{\overline{z_n} - 1}{g_n} \right| = \left\| \left( \frac{\overline{z_n} - 1}{g_n}\right) v_n \right\| \le 2 \Vert (KG(z_n) - KG(1))^*v_n\Vert \nonumber \\&+ \Vert (KG(z_n))^*v_n \Vert \le {\varGamma }, \end{aligned}$$

for some constant ${\varGamma } > 0$. Therefore, the sequence $(\tfrac{\overline{z_n} - 1}{g_n})_{n \in \mathbb {N}}$ is bounded and hence has a convergent subsequence, which we pass to without relabelling, and denote the limit by $l^\infty$. Taking the limit $n \rightarrow \infty$ in (8.26) yields that

$$\begin{aligned} 0 \ge \Vert l^\infty v^\infty + (K G(1))^*v^\infty \Vert \ge 0, \end{aligned}$$

whence

8.28 $$\begin{aligned} (K G(1))^*v^\infty = -l^\infty v^\infty . \end{aligned}$$

In particular, as $v^\infty \ne 0$ we conclude from (8.28) that $- l^\infty \in \sigma ((K G(1))^*) \subseteq \mathbb {C}_0^+$, since

$$\begin{aligned} \sigma ((K G(1))^*) = \overline{\sigma (K G(1))} \subseteq \overline{\mathbb {C}_0^+} = \mathbb {C}_0^+. \end{aligned}$$

Therefore, $\mathrm{Re}\, (-l^\infty ) \ge 2\alpha >0$, for some $\alpha >0$ and hence there exists $N \in \mathbb {N}$ such that

8.29 $$\begin{aligned} \mathrm{Re}\, \frac{1- z_n}{g_n} = \mathrm{Re}\, \frac{1-\overline{ z_n}}{g_n} \ge \alpha >0, \quad n \in \mathbb {N}, \; n \ge N. \end{aligned}$$

Define $z_n^\prime = 1 + \mathrm {i}\, \mathrm{Im} \, z_n$ for $n \in \mathbb {N}$ and using (8.25) and $\vert z_n \vert =1$ we compute

8.30 $$\begin{aligned} \frac{\vert z_n^\prime - z_n \vert }{\vert 1 - z_n \vert } = \frac{1 - \mathrm{Re}\, z_n}{\sqrt{2(1-\mathrm{Re} z_n) + \vert z_n\vert ^2 -1}} = \frac{1}{\sqrt{2}}\sqrt{(1-\mathrm{Re} \,z_n)} \rightarrow 0, \quad \text {as }n \rightarrow \infty .\nonumber \\ \end{aligned}$$

Now for each $n \in \mathbb {N}$

$$\begin{aligned} \frac{1 - z_n^\prime }{g_n} = \frac{1-z_n}{g_n} + \frac{z_n - z_n^\prime }{1- z_n} \cdot \frac{1-z_n}{g_n}, \end{aligned}$$

and thus

8.31 $$\begin{aligned} \mathrm{Re}\, \frac{1 - z_n^\prime }{g_n}&= \mathrm{Re}\, \left( \frac{1-z_n}{g_n} + \frac{z_n - z_n^\prime }{1- z_n} \cdot \frac{1-z_n}{g_n}\right) \nonumber \\&= \mathrm{Re}\, \left( \frac{1-z_n}{g_n} \right) + \mathrm{Re}\, \left( \frac{z_n - z_n^\prime }{1- z_n} \cdot \frac{1-z_n}{g_n}\right) \nonumber \\&\ge \mathrm{Re}\, \left( \frac{1-z_n}{g_n} \right) - \left| \frac{z_n - z_n^\prime }{1- z_n} \right| \cdot \left| \frac{1-z_n}{g_n}\right| . \end{aligned}$$

Inserting (8.27), (8.29) and (8.30) into (8.31) implies that

$$\begin{aligned} \liminf _{n \rightarrow \infty } \left( \mathrm{Re}\, \frac{1 - z_n^\prime }{g_n} \right) \ge \frac{\alpha }{2} >0, \end{aligned}$$

which is a contradiction, since by construction

$$\begin{aligned} \mathrm{Re}\, \frac{1 - z_n^\prime }{g_n} = 0, \quad n \in \mathbb {N}. \end{aligned}$$

The inequality (8.18) is sufficient for us to invoke a small-gain proof. From (8.15) we have that for each $t \in \mathbb {N}$

8.32 $$\begin{aligned} \xi (t)= & {} \mathcal {A}^t \xi (0) + \sum _{j=0}^{t-1} \mathcal {A}^{t-1 -j} \mathcal {B}{\varPsi } (\mathcal {C}\xi (j)) \nonumber \\ \Rightarrow \mathcal {C}\xi (t)= & {} \mathcal {C}\mathcal {A}^t \xi (0) + \sum _{j=0}^{t-1} \mathcal {C}\mathcal {A}^{t-1 -j} \mathcal {B}{\varPsi } (\mathcal {C}\xi (j)) . \end{aligned}$$

Since $r(\mathcal {A}) <1$, (and $\mathcal {B}$ and $\mathcal {C}$ are bounded) it follows that the sequences *a* and *h* with terms $a(t) = \mathcal {C}\mathcal {A}^t$ and $h(t) = \mathcal {C}\mathcal {A}^t \mathcal {B}$, $t \in \mathbb {N}_0$, respectively, both belong to $\ell ^2$. Therefore, we estimate from (8.32) for $T \in \mathbb {N}$

8.33 $$\begin{aligned} \Vert \mathcal {C}\xi \Vert _{\ell ^2(0,T)}&\le \Vert a \Vert _{\ell ^2(0,T)} \cdot \Vert \xi (0) \Vert + \Vert h *{\varPsi }(\mathcal {C}\xi )\Vert _{\ell ^2(0,T-1)} \nonumber \\&\le \Vert a \Vert _{\ell ^2(0,T)} \cdot \Vert \xi (0) \Vert + \Vert \mathcal {G}_g \Vert _\infty \cdot \Vert {\varPsi }(\mathcal {C}\xi ) \Vert _{\ell ^2(0,T)}, \nonumber \\&\le \Vert a \Vert _{\ell ^2(0,T)} \cdot \Vert \xi (0) \Vert + \Vert \mathcal {G}_g \Vert _\infty \cdot \Vert \mathcal {C}\xi \Vert _{\ell ^2(0,T)}, \end{aligned}$$

where we have used the linear bound (8.12) for ${\varPsi }$ and the convolution bound (7.11). Invoking the estimate (8.18) we rearrange (8.33)

8.34 $$\begin{aligned} \Vert \mathcal {C}\xi \Vert _{\ell ^2(0,T)} \le \frac{\Vert a \Vert _{\ell ^2(0,T)} \cdot \Vert \xi (0) \Vert }{1 - \Vert \mathcal {G}_g \Vert _\infty } \le \frac{\Vert a \Vert _{\ell ^2} \cdot \Vert \xi (0) \Vert }{1 - \Vert \mathcal {G}_g \Vert _\infty }. \end{aligned}$$

The bound (8.34) holds for every $T \in \mathbb {N}$, and we hence conclude that ${\tilde{x}}_c = \mathcal {C}\xi \in \ell ^2$ and thus claim (a) holds, that is,

8.35 $$\begin{aligned} {\tilde{x}}_c(t) \rightarrow 0, \quad \text {as }t \rightarrow \infty \Rightarrow x_c(t) \rightarrow \mathrm{sat}\,(v) = G(1)^{-1} r, \quad \text {as }t \rightarrow \infty . \end{aligned}$$

From (8.15) it follows that ${\tilde{x}}$ has dynamics

$$\begin{aligned} {\tilde{x}}(t+1) = A{\tilde{x}}(t) + B \widetilde{\mathrm{sat}}\,({\tilde{x}}_c(t)), \quad {\tilde{x}}(0) = x^0 - x^\infty , \quad t \in \mathbb {N}_0\, \end{aligned}$$

whence, as $r(A) <1$ and by (8.35)

$$\begin{aligned} {\tilde{x}}(t) = A^t {\tilde{x}}(0) + \sum _{j=0}^{t-1} A^{t-1 -j} B \widetilde{\mathrm{sat}}\,({\tilde{x}}_c(t)) \rightarrow 0, \quad \text {as }t \rightarrow \infty , \end{aligned}$$

proving parts (b) and (c). $\square$

### *Proof of Corollary 4.7*

We only prove the result for (PI1aw), as the proof for (PI2aw) is very similar and is omitted. With $F_1$ chosen as in the statement of the result, it follows immediately from inspection of (PI1aw) that

8.36 $$\begin{aligned} x(t+1)&= Ax(t) + Bu(t) = Ax(t) - BF_1 x(t) + B \mathrm{sat}\,(x_c(t)) \nonumber \\&= (A-BF_1)x(t) + B \mathrm{sat}\,(x_c(t)), \quad x(0) = x^0, \quad t \in \mathbb {N}_0, \end{aligned}$$

so that (PI1aw) specified by (*A*, *B*, *C*) is in fact an instance of (Iaw) specified by $(A_1,B,C)$. By assumption $A_1 := A-BF_1 \in \mathbb {R}^{n \times n}_+$, so that $(A_1,B,C)$ satisfy (4.1), (A1) and (A2) hold for $(A_1,B,C)$ and thus the result follows by applying Theorem 4.6 to (Iaw) specified by $(A_1,B,C)$. $\square$

### *Proof of Proposition 4.9*

The proof is based on that of Theorem 4.6. Introducing the shifted co-ordinates ${\tilde{x}}$ and ${\tilde{x}}_c$ as in (8.10a) and (8.10b), respectively, then the disturbed feedback system can be written as

8.37 $$\begin{aligned}&\begin{bmatrix}{\tilde{x}} (t+1) \\ {\tilde{x}}_c (t+1) \end{bmatrix} = \begin{bmatrix}A&B \\ -gKC&I \end{bmatrix}\begin{bmatrix}{\tilde{x}} (t) \\ {\tilde{x}}_c (t) \end{bmatrix} - \begin{bmatrix}B \\ E \end{bmatrix}{\varPsi }\left( \begin{bmatrix}0&I \end{bmatrix}\begin{bmatrix}{\tilde{x}} (t) \\ {\tilde{x}}_c (t) \end{bmatrix}\right) + \begin{bmatrix} d_1(t) \\ -gK d_2(t) \end{bmatrix}, \nonumber \\&\quad t \in \mathbb {N}_0, \end{aligned}$$

which after introducing

8.38 $$\begin{aligned}&\xi := \begin{bmatrix}{\tilde{x}} \\ {\tilde{x}}_c \end{bmatrix}, \quad \mathcal {D}:= \begin{bmatrix}d_1 \\ -gKd_2 \end{bmatrix}, \quad \mathcal {A}:= \begin{bmatrix}A&B \\ -gKC&I \end{bmatrix}, \nonumber \\&\quad \mathcal {B}:= - \begin{bmatrix}B \\ E \end{bmatrix} \quad \text {and} \quad \mathcal {C}:= \begin{bmatrix}0&I \end{bmatrix}, \end{aligned}$$

we rewrite as

8.39 $$\begin{aligned} \xi (t+1) = \mathcal {A}\xi (t) + \mathcal {B}{\varPsi }(\mathcal {C}\xi (t)) + \mathcal {D}(t), \quad \xi (0) = \xi _0, \quad t \in \mathbb {N}_0. \end{aligned}$$

The solution $\xi$ of (8.39) can be expressed as

8.40 $$\begin{aligned} \xi (t) = \mathcal {A}^t \xi (0) + \sum _{j=0}^{t-1} \mathcal {A}^{t-1-j} \mathcal {B}\psi (\mathcal {C}\xi (j)) + \sum _{j=0}^{t-1} \mathcal {A}^{t-1-j} \mathcal {D}(j), \quad t \in \mathbb {N}. \end{aligned}$$

From the proof of Theorem 4.6 there exists $g^*>0$ such that for all $g \in (0, g^*)$

$$\begin{aligned} r(\mathcal {A}) <1 \quad \text {and} \quad \Vert \mathcal {G}_{g} \Vert _\infty = \sup _{z \in \mathbb {T}} \Vert \mathcal {C}(zI-\mathcal {A})^{-1} \mathcal {B}\Vert < 1, \end{aligned}$$

(see (8.16) and (8.18), respectively). Therefore, by continuity for each $g \in (0, g^*)$ there exists $\mu >1$ such that

8.41 $$\begin{aligned} r(\mu \mathcal {A}) = \mu r(\mathcal {A}) <1 \quad \text {and} \quad \Vert \mathcal {G}_{g,\mu } \Vert _\infty = \sup _{z \in \mathbb {T}} \Vert \mathcal {C}(zI-\mu \mathcal {A})^{-1} \mathcal {B}\Vert <1. \end{aligned}$$

Multiplying (8.40) by $\mu ^{t-1}$ and setting $\eta (t) := \mu ^{t-1} \xi (t)$, $t \in \mathbb {N}_0$ gives

8.42 $$\begin{aligned} \eta (t)\! =\! (\mu \mathcal {A})^t \eta (0) \!+\! \sum _{j=0}^{t-1} (\mu \mathcal {A})^{t-1-j} \mathcal {B}\psi (\mathcal {C}\xi (j))\mu ^j \!+\! \sum _{j=0}^{t-1} (\mu \mathcal {A})^{t-1-j} \mathcal {D}(j)\mu ^j, \quad t \!\in \! \mathbb {N},\nonumber \\ \end{aligned}$$

and applying $\mathcal {C}$ to (8.42) produces

8.43 $$\begin{aligned} \mathcal {C}\eta (t)= & {} \mathcal {C}(\mu \mathcal {A})^t \eta (0) + \sum _{j=0}^{t-1} \mathcal {C}(\mu \mathcal {A})^{t-1-j} \mathcal {B}\psi (\mathcal {C}\xi (j))\mu ^j\nonumber \\&+ \sum _{j=0}^{t-1} \mathcal {C}(\mu \mathcal {A})^{t-1-j} \mathcal {D}(j)\mu ^j, \quad t \in \mathbb {N}. \end{aligned}$$

Introduce the sequences $h_\mu$, $a_\mu , s(\mu , \xi )$ and $\mathcal {D}_\mu$ with respective terms

$$\begin{aligned} h_\mu (t)= & {} \mathcal {C}(\mu \mathcal {A})^t \mathcal {B}, \quad a_\mu (t) = \mathcal {C}(\mu \mathcal {A})^t, \\ s(\mu , \xi )(t)= & {} {\varPsi }(\mathcal {C}\xi (t))\mu ^t, \quad \mathcal {D}_\mu (j) = \mathcal {D}(j)\mu ^j, \quad t \in \mathbb {N}_0. \end{aligned}$$

The property $r(\mu A)<1$ from (8.41) implies that $a_\mu , h_\mu \in \ell ^1 \cap \ell ^2$. We estimate (8.43) in a similar manner to as in (8.33), yielding that

8.44 $$\begin{aligned} \Vert \mathcal {C}\eta \Vert _{\ell ^2(0,t)}&\le \Vert a_\mu \Vert _{\ell ^2(0,t)} \cdot \Vert \eta (0) \Vert + \Vert h_\mu *s(\mu , \xi )\Vert _{\ell ^2(0,t-1)} + \Vert h_\mu *\mathcal {D}_\mu \Vert _{\ell ^2(0,t-1)} \nonumber \\&\le c_1 \Vert \xi (0) \Vert \!+\! \Vert \mathcal {G}_{g,\mu } \Vert _\infty \cdot \Vert s(\mu , \xi ) \Vert _{\ell ^2(0,t)} \!+\! \Vert h_\mu \Vert _{\ell ^1(0,t-1)} \cdot \Vert \mathcal {D}_\mu \Vert _{\ell ^2(0,t-1)}, \nonumber \\&\le c_1 \Vert \xi (0) \Vert + \Vert \mathcal {G}_{g,\mu } \Vert _\infty \cdot \Vert \mathcal {C}\eta \Vert _{\ell ^2(0,t)} + c_2\Vert \mathcal {D}_\mu \Vert _{\ell ^2(0,t-1)}, \quad t \in \mathbb {N}, \end{aligned}$$

where $c_1 = \Vert a_\mu \Vert _2, c_2 = \Vert h_\mu \Vert _1$, we have used the convolution estimate (7.6), the linear estimate (8.12) and the definition of $\eta$. Rearranging (8.44) gives

8.45 $$\begin{aligned} \Vert C \eta \Vert _{\ell ^2(0,t)} \le c_3 \Vert \eta (0) \Vert + c_4 \Vert \mathcal {D}_\mu \Vert _{\ell ^2(0,t-1)}, \quad t \in \mathbb {N}, \end{aligned}$$

with constants

$$\begin{aligned} c_3 = \frac{c_1}{1 - \Vert \mathcal {G}_{g,\mu } \Vert _\infty }, \quad c_4 = \frac{c_2}{1 - \Vert \mathcal {G}_{g,\mu } \Vert _\infty }. \end{aligned}$$

Taking norms in (8.42) gives that

8.46 $$\begin{aligned} \Vert \eta (t) \Vert&\le \Vert (\rho A)^t \eta (0) \Vert + \left\| \sum _{j=0}^{t-1} (\mu \mathcal {A})^{t-1-j} \mathcal {B}\psi (\mathcal {C}\xi (j))\mu ^j \right\| \nonumber \\&\quad + \left\| \sum _{j=0}^{t-1} (\mu \mathcal {A})^{t-1-j} \mathcal {D}(j)\mu ^j \right\| , \quad t \in \mathbb {N}. \end{aligned}$$

The second and third terms on the right hand side of (8.46) are convolutions, which we bound from above using (7.7) to give

$$\begin{aligned} \Vert \eta (t) \Vert&\le c_5 \Vert \eta (0) \Vert + \left( \sum _{k=0}^{t-1} \Vert (\mu \mathcal {A})^k \mathcal {B}\Vert ^2 \right) ^{\tfrac{1}{2}} \cdot \Vert s(\mu ,\xi )\Vert _{\ell ^2(0,t-1)}\\&\quad + \left( \sum _{k=0}^{t-1} \Vert (\mu \mathcal {A})^k \Vert ^2 \right) ^{\tfrac{1}{2}} \cdot \Vert \mathcal {D}_\mu \Vert _{\ell ^2(0,t-1)} \end{aligned}$$

where $c_5 := \Vert a_\mu \Vert _\infty$. As $r(\mu \mathcal {A})<1$ and $\mathcal {B}$ is bounded, there exist constants $c_6$ and $c_7$ such that

8.47 $$\begin{aligned} \Vert \eta (t) \Vert&\le c_5 \Vert \eta (0) \Vert + c_6 \Vert s(\mu , \xi ) \Vert _{\ell ^2(0,t-1)} + c_7 \Vert \mathcal {D}_\mu \Vert _{\ell ^2(0,t-1)}\nonumber \\&\le c_5 \Vert \eta (0) \Vert + c_6 \Vert \mathcal {C}\eta \Vert _{\ell ^2(0,t)} + c_7 \Vert \mathcal {D}_\mu \Vert _{\ell ^2(0,t-1)}\nonumber \\&\le \underbrace{(c_5 + c_6 c_3)}_{=:c_8} \Vert \eta (0) \Vert + \underbrace{(c_7+ c_6c_4)}_{=:c_9} \Vert \mathcal {D}_\mu \Vert _{\ell ^2(0,t-1)}, \quad t \in \mathbb {N}, \end{aligned}$$

where we have inserted (8.45). We compute that

$$\begin{aligned} \Vert \mathcal {D}_\mu \Vert _{\ell ^2(0,t-1)}&= \left( \sum _{j=0}^{t-1} \Vert \mu ^j \mathcal {D}(j) \Vert ^2 \right) ^{\tfrac{1}{2}} \le \left( \sum _{j=0}^{t-1} \mu ^{2j} \right) ^{\tfrac{1}{2}}\Vert \mathcal {D}\Vert _{\ell ^\infty (0,t-1)}\\&= \left( \frac{\mu ^2(\mu ^{2t} -1)}{\mu ^2 -1}\right) ^{\tfrac{1}{2}}\Vert \mathcal {D}\Vert _{\ell ^\infty (0,t-1)} \\&\le c_{10} \mu ^t \Vert \mathcal {D}\Vert _{\ell ^\infty (0,t-1)}, \quad t \in \mathbb {N}, \end{aligned}$$

which, when substituted into (8.47) produces

$$\begin{aligned} \Vert \eta (t) \Vert \le c_8 \Vert \eta (0) \Vert + c_9 c_{10} \mu ^t \Vert \mathcal {D}\Vert _{\ell ^\infty (0,t-1)}, \quad t \in \mathbb {N}. \end{aligned}$$

Recalling that $\eta (t) = \mu ^{t-1}\xi (t)$ we obtain

$$\begin{aligned} \Vert \xi (t) \Vert&\le c_8 \mu ^{-t} \Vert \xi (0) \Vert + c_9 c_{10} \mu \Vert \mathcal {D}\Vert _{\ell ^\infty (0,t-1)} \\&\le c_8 \mu ^{-t} \Vert \xi (0) \Vert + c_{11}\Vert d_1 \Vert _{\ell ^\infty (0,t-1)} + c_{12}\Vert d_2 \Vert _{\ell ^\infty (0,t-1)}, \quad t \in \mathbb {N}, \end{aligned}$$

from the definition of $\mathcal {D}$ in (8.38). We have established the first two estimates in (4.8). To acquire the estimate for $y(t)-r$, we repeat the above calculation from (8.46), but now estimate $$y(t) -r = \left[ {\begin{matrix}C&0 \end{matrix}}\right] \xi (t)$$ instead of $\xi (t)$. The proof is the same as above, as $$\begin{bmatrix}C&0 \end{bmatrix}$$ is bounded. In summary, we have established (4.8) with $\gamma = \tfrac{1}{\mu } \in (0,1)$ and constants $M_0$, $M_1$ and $M_2$ relabelled from the $c_j$ constants appropriately. $\square$

### *Proof of Corollary 4.10*

The proof of the result borrows heavily from the proof of (4.8) in Proposition 4.9. For given $f_1^\infty$ and $d_2^\infty$ let *r* and $u_+$ be as in (4.9), and note that

$$\begin{aligned} u_+ = \mathrm{sat}\,(v), \end{aligned}$$

for some $v \in \mathbb {R}^m_+$ (not necessarily unique). Define

8.48 $$\begin{aligned} w^\infty : = (I-A)^{-1}B(\mathrm{sat}\,(v) + f_1^\infty ), \end{aligned}$$

Define the shifted co-ordinates ${\tilde{x}}$ and ${\tilde{x}}_c$ by

$$\begin{aligned} {\tilde{x}} (t) := x(t) - w^\infty , \quad {\tilde{x}}_c (t) := x_c(t) - \mathrm{sat}\,(v), \quad t \in \mathbb {N}_0. \end{aligned}$$

An elementary sequence of calculations shows that ${\tilde{x}}$ and ${\tilde{x}}_c$ have dynamics given by

8.49 $$\begin{aligned} \begin{bmatrix}{\tilde{x}} (t+1) \\ {\tilde{x}}_c (t+1) \end{bmatrix}= & {} \begin{bmatrix}A&B \\ -gKC&I \end{bmatrix}\begin{bmatrix}{\tilde{x}} (t) \\ {\tilde{x}}_c (t) \end{bmatrix} - \begin{bmatrix}B \\ E \end{bmatrix}{\varPsi }\left( \begin{bmatrix}0&I \end{bmatrix}\begin{bmatrix}{\tilde{x}} (t) \\ {\tilde{x}}_c (t) \end{bmatrix}\right) \nonumber \\&+ \begin{bmatrix}B (f_1(t) - f_1^\infty ) \\ -gK (d_2(t) - d_2^\infty ) \end{bmatrix}, \quad t \in \mathbb {N}_0, \end{aligned}$$

which can be written as (8.39) with

8.50 $$\begin{aligned} \mathcal {D}(t) = \begin{bmatrix}B (f_1(t) - f_1^\infty ) \\ -gK (d_2(t) - d_2^\infty ) \end{bmatrix}, \quad t \in \mathbb {N}_0. \end{aligned}$$

In deriving Proposition 4.9 we established the existence of a $g^*>0$ such that for all $g \in (0,g^*)$ and all initial states $(x^0, x_c^0) \in \mathbb {R}^n_+ \times \mathbb {R}^m_+$, there exists $\mu >1$ and constants $C_1,C_2>0$ such that the solution $$\xi = \left[ {\begin{matrix}x - w^\infty \\ x_c - \widetilde{\mathrm{sat}}\,(v) \end{matrix}}\right] $$ of (8.39) satisfies the estimate

8.51 $$\begin{aligned} \Vert \xi (t) \Vert \le C_1 \mu ^{-t} \Vert \xi (0) \Vert + C_2 \mu \Vert \mathcal {D}\Vert _{\ell ^\infty (0,t-1)}, \quad t \in \mathbb {N}. \end{aligned}$$

Since $\mathcal {D}$ in (8.50) is convergent, it is bounded and hence the inequality (8.51) implies that $\xi$ is bounded. A straightforward time-invariance argument yields that for every $T \in \mathbb {N}_0$

8.52 $$\begin{aligned} \Vert \xi (t+T) \Vert \le C_1 \mu ^{-t} \Vert \xi (T) \Vert + C_2 \mu \Vert \mathcal {D}\Vert _{\ell ^\infty (T,T+t-1)}, \quad t \in \mathbb {N}. \end{aligned}$$

By construction $\mathcal {D}(t) \rightarrow 0$ as $t \rightarrow \infty$, which coupled with the boundedness of $\xi$ and (8.52) implies that $\xi (t) \rightarrow 0$ as $t \rightarrow \infty$, proving the corollary. $\square$

### *Proof of Lemma 4.11*

The proof is elementary using the follow identity

$$\begin{aligned} (V+W)^{-1} = V^{-1} - (V+W)^{-1}WV^{-1}, \end{aligned}$$

for both *V* and $V+W$ boundedly invertible, which itself follows from rearranging $(V+W)^{-1}(V +W) =I$. As such, for $z \in \mathbb {C}\cap \rho (A) \cap \rho ( {\hat{A}})$

8.53 $$\begin{aligned} (zI -A)^{-1}= & {} ((zI- {\hat{A}}) - {\Delta } A)^{-1}\nonumber \\= & {} (zI- {\hat{A}})^{-1} + ((zI- {\hat{A}}) - {\Delta } A)^{-1} ({\Delta } A) (zI- {\hat{A}})^{-1}. \end{aligned}$$

The result now follows by multiplying (8.53) by $C = {\hat{C}} + {\Delta } C$ and $B = {\hat{B}} + {\Delta } B$ on the left and right hand sides respectively, expanding and collecting together terms as suggested. $\square$

### *Proof of Lemma 4.12*

The proof makes use of the complex stability radius developed by Hinrichsen and Pritchard (1986a, b). For given $Q \in \mathbb {C}^{m \times m}$ with $\sigma (Q) \subseteq \mathbb {C}_0^+$, then clearly $\sigma (-Q) \subseteq \mathbb {C}_0^-$, with $\mathbb {C}_0^-$ the open left-half complex plane. For assumption (A2) we require that

$$\begin{aligned} -KG(1) = -K{\hat{G}}(1) - K {\Delta } G(1) = -Q - K {\Delta } G(1), \end{aligned}$$

also has spectrum contained in $\mathbb {C}_0^-$. Viewing $-K {\Delta } G(1)$ as a structured perturbation to $-Q$, by Hinrichsen and Pritchard (1986b, Proposition 2.1) it follows that $\sigma (-KG(1))\subseteq \mathbb {C}_0^-$ if

$$\begin{aligned} \Vert - K {\Delta } G(1) \Vert = \Vert K {\Delta } G(1)\Vert < \frac{1}{\sup _{\omega \in \mathbb {R}}\Vert (\omega \mathrm {i}+ Q)^{-1} \Vert }, \end{aligned}$$

which, noting that $K = Q {\hat{G}}(1)^{-1}$, is (4.12). In the case that $Q=I$ then

$$\begin{aligned} \sup _{\omega \in \mathbb {R}}\Vert (\omega \mathrm {i}+ Q)^{-1} \Vert = \sup _{\omega \in \mathbb {R}} \Vert (\omega \mathrm {i}+ 1)^{-1}I \Vert = \sup _{\omega \in \mathbb {R}}\frac{1}{\vert \omega \mathrm {i}+ 1\vert } =1, \end{aligned}$$

yielding one ingredient of the inequality (4.13). When $Q = I$, so that $K= {\hat{G}}(1)^{-1}$, then we also note that

$$\begin{aligned} -G(1)K = -{\hat{G}}(1)K - {\Delta } G(1)K = -I - {\Delta } G(1)K, \end{aligned}$$

and thus $\sigma (-G(1)K) \subseteq \mathbb {C}_0^-$ if

$$\begin{aligned} \Vert - {\Delta } G(1)K \Vert = \Vert {\Delta } G(1)K\Vert < \frac{1}{\sup _{\omega \in \mathbb {R}}\Vert (\omega \mathrm {i}+ I)^{-1} \Vert } = 1, \end{aligned}$$

which is the second ingredient in establishing (4.13). Here we have used that $\sigma (-G(1)K) \subseteq \mathbb {C}_0^-$ if, and only if, $\sigma (-KG(1)) \subseteq \mathbb {C}_0^-$, which follows from the fact that the non-zero eigenvalues of $-KG(1)$ are precisely equal to those of $-G(1)K$. That (4.14) is sufficient for (4.13) follows immediately from the fact that the matrix 2–norm satisfies

$$\begin{aligned} \Vert {\Delta } G(1) {\hat{G}}(1)^{-1} \Vert \, , \Vert {\hat{G}}(1)^{-1} {\Delta } G(1) \Vert < \Vert {\hat{G}}(1)^{-1} \Vert \cdot \Vert {\Delta } G(1)\Vert . \end{aligned}$$

$$\square $$

### *Proof of Lemma 4.13*

If (a) holds then we simply estimate

$$\begin{aligned} \Vert (I\!+\!X)^{-1}X \Vert&\le \Vert (I+X)^{-1} \Vert \cdot \Vert X \Vert \!=\! \left\| \sum _{j \in \mathbb {N}_0} (-X)^j \right\| \cdot \Vert X \Vert \!\le \! \left( \sum _{j \in \mathbb {N}_0} \Vert X \Vert ^j\right) \cdot \Vert X \Vert \\&= \frac{\Vert X \Vert }{1 - \Vert X\Vert } <1 \iff \Vert X \Vert < \frac{1}{2}, \end{aligned}$$

as required. We claim that (b) implies that $X + X^*\succcurlyeq 0$, where $P \succcurlyeq 0$ or $0 \preccurlyeq P$ both denote positive definiteness of *P* (as opposed to the usual $P \ge 0$ or $0 \le P$, which in this manuscript denotes componentwise nonnegativity of *P*). Denote $Y = (I-X)(I+X)^{-1}$, and note that (b) implies that $\Vert Y \Vert ^2 \le 1$ or, in other words, $I - Y^*Y \succcurlyeq 0$. From here we compute that

8.54 $$\begin{aligned} 0 \preccurlyeq I - Y^*Y&= I - (I+X)^{-*}(I-X)^*(I-X)(I+X)^{-1} \nonumber \\&= (I+X)^{-*} [ (I+X)^*(I+X) - (I-X)^*(I-X) ] (I+X)^{-1} \nonumber \\&= 2(I+X)^{-*} [ X + X^*] (I+X)^{-1} \nonumber \\ \Rightarrow 0&\preccurlyeq X+X^*, \end{aligned}$$

as claimed. The inequality $\Vert X \Vert \le 1$ implies that $\Vert X^*\Vert \le 1$ and thus, letting $\mathcal {H}$ denote the Hilbert space on which *X* is defined

8.55 $$\begin{aligned} \langle X^*v , X^*v \rangle \le \langle v, v \rangle = \Vert v \Vert ^2, \quad \forall v \in \mathcal {H}. \end{aligned}$$

We combine (8.54) and (8.55) and estimate for $v \in \mathcal {H}$ and $\rho ^2 \in (\tfrac{1}{2}, 1)$

$$\begin{aligned} 0&\le (2 \rho ^2 -1) \langle v, v \rangle + \rho ^2 \langle (X+ X^*) v,v \rangle \nonumber \\&\le \rho ^2 \langle v, v \rangle + (\rho ^2 -1) \langle X^*v, X^*v \rangle + \rho ^2\langle (X+ X^*) v,v \rangle \nonumber \\&\Rightarrow \langle X^*v, X^*v \rangle \le \rho ^2 \langle (I +X)^*v, (I +X)^*v \rangle , \end{aligned}$$

and taking $u = (I+X)^*v \in \mathcal {H}$ yields that

8.56 $$\begin{aligned} \langle X^*(I +X)^{-*} u, X^*(I +X)^{-*} u \rangle&\le \rho ^2 \langle u, u \rangle \Rightarrow \Vert X^*(I +X)^{-*} u \Vert \le \rho \Vert u \Vert . \end{aligned}$$

Since $v \in \mathcal {H}$ and hence $u \in \mathcal {H}$ was arbitrary, we conclude from (8.56) that

$$\begin{aligned} \Vert (I+X)^{-1} X \Vert = \Vert X^*(I +X)^{-*} \Vert = \sup _{ \begin{array}{c} u \in \mathcal {H}\\ u \ne 0 \end{array}} \frac{\Vert X^*(I +X)^{-*} u \Vert }{\Vert u \Vert } \le \rho <1, \end{aligned}$$

as required. $$\square $$

The next lemma is a technical result that prepares the proof of Corollary 4.15. The lemma demonstrates that the assumptions of Corollary 4.15 are sufficient for the (unknown) transfer function $\mathcal {G}_g$ associated with the feedback system (Iaw) to satisfy

8.57 $$\begin{aligned} \Vert \mathcal {G}_g \Vert _\infty <1, \end{aligned}$$

for all perturbations ${\Delta } G$ that are not too large in norm. Once (8.57) is established, then the proof of Corollary 4.15 is identical to the latter part of the proof of Theorem 4.6. Establishing (8.57) is achieved in part by choosing $g >0$ sufficiently small, and hence we obtain a low-gain result. However, the value of $\mathcal {G}_g(1)$ is independent of *g*, indeed

$$\begin{aligned} \Vert \mathcal {G}_g \Vert _\infty \ge \Vert \mathcal {G}_g(1) \Vert = \Vert [I + {\hat{G}}(1) {\Delta } G(1) ]^{-1} \cdot [{\hat{G}}(1) {\Delta } G(1)] \Vert , \end{aligned}$$

and hence condition (4.17) (see also (8.58) below) is necessary for (8.57) to hold.

### **Lemma 6.1**

Suppose that $G = {\hat{G}} + {\Delta } G \in H^\infty$ where ${\hat{G}} \in H^\infty$ and $K \in \mathbb {R}^{m \times m}$ is such that *K* and ${\hat{G}}(1)$ satisfy $\sigma (K{\hat{G}}(1)) \subseteq \mathbb {C}_0^+$. Then, there exists $\rho \in (0,1)$, $M^*>0$ and $g^*>0$ (which depends on $M^*$) such that for all ${\Delta } G$ in (4.10) with

8.58 $$\begin{aligned} \Vert {\Delta } G \Vert _\infty < M^*\quad \text {and} \quad \Vert [I + {\hat{G}}(1)^{-1} {\Delta } G(1) ]^{-1} \cdot [{\hat{G}}(1)^{-1} {\Delta } G(1)] \Vert <1, \end{aligned}$$

and all $g \in (0,g^*)$, the function

$$\begin{aligned} z \mapsto \mathcal {G}_g(z) := ((z-1)I + gKG(z))^{-1} (gKG(z) - gK{\hat{G}}(1)), \quad z \in \mathbb {C}, \; \vert z \vert \ge 1, \end{aligned}$$

belongs to $H^\infty$ and

8.59 $$\begin{aligned} \Vert \mathcal {G}_g \Vert _\infty \le \rho . \end{aligned}$$

### *Proof*

First note that as $\sigma (K{\hat{G}}(1)) \subseteq \mathbb {C}_0^+$ is a finite set and eigenvalues depend continuously on bounded perturbations, we may choose ${\hat{N}} >0$ such that

8.60 $$\begin{aligned} \Vert {\Delta } G \Vert _\infty < {\hat{N}} \Rightarrow \sigma (KG(1)) \subseteq \mathbb {C}_\alpha ^+, \end{aligned}$$

for some $\alpha >0$. The spectrum condition (8.60) implies that $G(1)= {\hat{G}}(1) + {\Delta } G(1)$ is invertible (see Remark 3.2), as is ${\hat{G}}(1)$. Hence

$$\begin{aligned} I + {\hat{G}}(1)^{-1} {\Delta } G(1) = {\hat{G}}(1)^{-1} \cdot G(1), \end{aligned}$$

is also invertible, and thus the operator that appears in the second estimate in (8.58) is well-defined whenever $\Vert {\Delta } G \Vert _\infty < {\hat{N}}$.

We seek to establish the existence of ${\hat{M}}> 0$ and ${\hat{g}} >0$ such that for all ${\Delta } G$ with $\Vert {\Delta } G \Vert _\infty \le {\hat{M}}$ and for all $g \in (0, {\hat{g}})$ it follows that $\mathcal {G}_g \in H^\infty$. By inspection of $\mathcal {G}_g$, it suffices to prove that for ${\Delta } G$ and *g* as above

$$\begin{aligned} \mathbb {E}\cup \mathbb {T}\ni z \mapsto T(z) := (z-1)I + gKG(z), \end{aligned}$$

satisfies $T^{-1} \in H^\infty$, as then $\mathcal {G}_g \in H^\infty$ as the product of $H^\infty$ functions. Boundedness of *gKG* implies that

8.61 $$\begin{aligned} \Vert T(z) v \Vert \ge \kappa \Vert v \Vert , \quad \forall v \in \mathbb {C}^m, \; \forall z \in \mathbb {T}\cup \mathbb {E}, \; \vert z \vert \ge R, \end{aligned}$$

for some *R* sufficiently large. The function $T^{-1}$ is analytic if

$$\begin{aligned} \mathbb {T}\cup \mathbb {E}\ni z \mapsto \det (T(z)), \end{aligned}$$

has no zeros in $\mathbb {T}\cup \mathbb {E}$. The function $z \mapsto \det (T(z))$ is itself analytic, and thus can only have at most finitely many zeros (since by (8.61) it is not identically zero), and we seek to prove that it has none.

The spectrum condition (8.60) implies that *T*(*z*) is invertible at $z=1$ for any $g >0$ and ${\Delta } G$ with $\Vert {\Delta } G \Vert < {\hat{N}}$. Therefore, by continuity, there exists ${\hat{r}} >0$, ${\hat{M}}_1 >0$ (with ${\hat{M}}_1 < {\hat{N}}$) and ${\hat{g}}_1 > 0$ such that for all ${\Delta } G$ with $\Vert {\Delta } G \Vert _\infty \le {\hat{M}}_1$, and for all $g \in (0, {\hat{g}}_1)$

8.62 $$\begin{aligned} \det (T(z)) \ne 0, \quad \forall z \in B(1,{\hat{r}}), \end{aligned}$$

where $B(1,{\hat{r}})$ is the complex ball of radius ${\hat{r}}$ and centre one. Moreover, we estimate that

$$\begin{aligned} \Vert T(z) \Vert = \Vert (z-1)I + gKG(z)\Vert \ge \frac{{\hat{r}}}{2} - g \Vert K G(z)\Vert , \quad \forall z \in (\mathbb {E}\cup \mathbb {T}){\setminus } B\left( 1,\tfrac{{\hat{r}}}{2}\right) , \end{aligned}$$

and hence there exists $\varepsilon >0$, ${\hat{M}}_2 >0$ (again with ${\hat{M}}_2 < {\hat{N}}$) and ${\hat{g}}_2 >0$ such that for all ${\Delta } G$ with $\Vert {\Delta } G \Vert _\infty \le {\hat{M}}_2$, and for all $g \in (0, {\hat{g}}_2)$

8.63 $$\begin{aligned} \Vert T(z) \Vert \ge \varepsilon , \quad \forall z \in (\mathbb {E}\cup \mathbb {T}){\setminus } B\left( 1,\tfrac{{\hat{r}}}{2}\right) . \end{aligned}$$

The lower bound (8.63) implies that *T* is injective and thus invertible on $(\mathbb {E}\cup \mathbb {T}){\setminus } B\left( 1,\tfrac{{\hat{r}}}{2}\right)$. Taking ${\hat{M}} = \min \{ {\hat{M}}_1 , {\hat{M}}_2\}$ and ${\hat{g}} = \min \{ {\hat{g}}_1 , {\hat{g}}_2\}$ and combining (8.62) and (8.63) we have proven that there exists $\hat{M} >0$ and ${\hat{g}}>0$ such that for all ${\Delta } G$ with $\Vert {\Delta } G \Vert < {\hat{M}}$ and all $g \in (0, {\hat{g}})$ then $T^{-1} \in H^\infty$ and hence $\mathcal {G}_g \in H^\infty$ as well.

To prove the norm estimate (8.59) we argue by contradiction and suppose that the claim is false. Then, there exists sequences $(M_n)_{n \in \mathbb {N}}, (g_n)_{n \in \mathbb {N}} \subseteq (0, \infty )$ and $( {\Delta }_n G)_{n \in \mathbb {N}}$ such that $g_n, M_n \searrow 0$ as $n \rightarrow \infty$,

8.64 $$\begin{aligned} \Vert {\Delta }_n G \Vert _\infty < M_n \quad \text {and} \quad \Vert [I \!+\! {\hat{G}}(1)^{-1} {\Delta } G(1) ]^{-1} \cdot [{\hat{G}}(1)^{-1} {\Delta } G(1)] \Vert \!<\!1, \quad \forall n \in \mathbb {N},\nonumber \\ \end{aligned}$$

but

$$\begin{aligned} \Vert \mathcal {G}_{g_n} \Vert _\infty > 1-\frac{1}{n} , \end{aligned}$$

where for $$n \in \mathbb {N}$$

$$\begin{aligned} z \mapsto \mathcal {G}_{g_n}(z)= & {} ((z-1)I + g_nK({\hat{G}}(z) + {\Delta }_n G(z)) )^{-1} g_nK ({\hat{G}}(z) \\&+ {\Delta }_n G(z) - {\hat{G}}(1)), \quad z \in \mathbb {T}\cup \mathbb {E}. \end{aligned}$$

Arguing as in the proof of Theorem 4.6, particularly between (8.18) and (8.23), the above supposition can be expressed as the existence of $(z_n)_{n \in \mathbb {N}} \subseteq \mathbb {T}$ and $(v_n)_{n \in \mathbb {N}} \subseteq \mathbb {C}^m$, with $\Vert v_n \Vert = 1$ such that

8.65 $$\begin{aligned}&\Vert g_n({\hat{G}}(z_n) + {\Delta }_n G(z_n) - {\hat{G}}(1))^*K^*v_n \Vert \nonumber \\&\quad > \left( 1-\frac{1}{n}\right) \Vert [(\overline{z_n} - 1)I + g_n({\hat{G}}(z_n)\nonumber \\&\qquad + {\Delta }_n G(z_n))^*K^*] v_n \Vert , \quad \forall n \in \mathbb {N}. \end{aligned}$$

As the sequences $(z_n)_{n \in \mathbb {N}}$ and $(v_n)_{n \in \mathbb {N}}$ are bounded, we may pass to a subsequence, without relabelling, along which $(z_n)_{n \in \mathbb {N}}$ and $(v_n)_{n \in \mathbb {N}}$ converge to respective limits $z^\infty \in \mathbb {T}$ and $v^\infty \in \mathbb {C}^m$. We note that $\Vert v^\infty \Vert =1$ and thus $v^\infty \ne 0$. The norm estimate (8.64) certainly implies that the sequence $({\Delta }_n G(z_n))_{n \in \mathbb {N}}$ is bounded and so taking a limit $n \rightarrow \infty$ in (8.65) gives

$$\begin{aligned} 0 \ge \Vert (\overline{z^\infty } - 1) v^\infty \Vert = \vert z^\infty -1 \vert \cdot \Vert v^\infty \Vert \ge 0. \end{aligned}$$

We deduce that $z^\infty = 1 = \overline{z^\infty }$. Dividing both sides of (8.65) by $g_n>0$, we obtain the following estimates

8.66 $$\begin{aligned} \Vert ({\hat{G}}(z_n) + {\Delta }_n G(z_n) - {\hat{G}}(1))^*K^*v_n\Vert&> \!\left( 1-\frac{1}{n}\right) \left\| \left[ \frac{(\overline{z_n} - 1)}{g_n}I \!+\! (KG(z_n))^*\right] v_n \right\| ,\nonumber \\&\ge \frac{1}{2}\left\| \left[ \frac{\overline{z_n} - 1}{g_n}I + (KG(z_n))^*\right] v_n \right\| , \quad n \ge 2 \nonumber \\&\ge \frac{1}{2}\left\| \left( \frac{\overline{z_n} - 1}{g_n}\right) v_n \right\| - \frac{1}{2}\Vert (KG(z_n))^*v_n \Vert , \end{aligned}$$

so that

8.67 $$\begin{aligned} 0\le & {} \left| \frac{\overline{z_n} - 1}{g_n} \right| = \left\| \left( \frac{\overline{z_n} - 1}{g_n}\right) v_n \right\| \nonumber \\\le & {} 2 \Vert ({\hat{G}}(z_n) + {\Delta }_n G(z_n) - {\hat{G}}(1))^*K^*v_n\Vert + \Vert (KG(z_n))^*v_n \Vert \le {\varGamma }, \end{aligned}$$

for some constant ${\varGamma } > 0$. Therefore, the sequence $(\tfrac{\overline{z_n} - 1}{g_n})_{n \in \mathbb {N}}$ is bounded and hence has a convergent subsequence, which we pass to without relabelling, and denote the limit by $l^\infty$.

We note that the norm estimate (8.64) implies that

$$\begin{aligned} {\Delta }_n G(z_n) \rightarrow 0, \quad \text {as }n \rightarrow \infty , \end{aligned}$$

and so taking the limit $n \rightarrow \infty$ in (8.66) yields that

$$\begin{aligned} 0 \ge \Vert l^\infty v^\infty + (K{\hat{G}}(1))^*v^\infty \Vert \ge 0, \end{aligned}$$

whence

8.68 $$\begin{aligned} (K{\hat{G}}(1))^*v^\infty = -l^\infty v^\infty . \end{aligned}$$

In particular, as $v^\infty \ne 0$ we conclude from (8.68) that $- l^\infty \in \sigma ((K{\hat{G}}(1))^*) \subseteq \mathbb {C}_0^+$, since

$$\begin{aligned} \sigma ((K{\hat{G}}(1))^*) = \overline{\sigma (K{\hat{G}}(1))} \subseteq \overline{\mathbb {C}_0^+} = \mathbb {C}_0^+. \end{aligned}$$

Therefore, $\mathrm{Re}\, (-l^\infty ) \ge 2\alpha >0$, for some $\alpha >0$ and hence there exists $N \in \mathbb {N}$ such that

8.69 $$\begin{aligned} \mathrm{Re}\, \frac{1- z_n}{g_n} = \mathrm{Re}\, \frac{1-\overline{ z_n}}{g_n} \ge \alpha >0, \quad n \in \mathbb {N}, \; n \ge N. \end{aligned}$$

However, $z_n \in \mathbb {T}$ and if $z_n =1$ for any $n \in \mathbb {N}$ with $n \ge N$ then $\mathrm{Re}\, \tfrac{1- z_n}{g_n} =0$, contradicting the uniformly positive estimate (8.69). Therefore, it suffices to suppose that

8.70 $$\begin{aligned} \mathrm{Re}\, z_n <1, \quad \forall n \in \mathbb {N}. \end{aligned}$$

The proof now finishes identically to that of Theorem 4.6, arguing from the line after (8.29). $\square$

### *Proof of Corollary 4.15*

The hypotheses of the corollary ensure that Lemma 6.1 applies and therefore for $g \in (0, g^*)$ the estimate (8.57) (or (8.59)) holds. The proof of the corollary is now the same as that of Theorem 4.6, following from the paragraph preceding Eq. (8.32). $\square$

## Footnotes

1 Quotes taken from http://mpe2013.org/about-mpe2013/.

2 Assumption (H) was labelled (A) in Guiver et al. (2014), which has been changed to (H) to avoid confusion with (A1) and (A2).

3 Where $\Vert G \Vert _\infty := \sup _{\mathrm{Re} \, z > 0} \Vert G(z)\Vert _2$ denotes the norm of the Hardy space $H^\infty = H^\infty (\mathbb {C}_0^+, \mathbb {C}^{m \times m})$ when $G \in H^\infty$ see, for example, Partington (2004, Definition 1.4.2).
